# Neuronal APOE4-induced early hippocampal network hyperexcitability in Alzheimer’s disease pathogenesis

**DOI:** 10.1038/s43587-026-01096-0

**Published:** 2026-04-03

**Authors:** Dennis R. Tabuena, Sung-Soo Jang, Brian Grone, Oscar Yip, Emily A. Aery Jones, Jessica Blumenfeld, Zherui Liang, Rajkamalpreet S. Mann, Yaqiao Li, Deanna Necula, Nicole Koutsodendris, Antara Rao, Leonardo Ding, Alex R. Zhang, Yanxia Hao, Qin Xu, Seo Yeon Yoon, Samuel De Leon, Yadong Huang, Misha Zilberter

**Affiliations:** 1 Gladstone Institute of Neurological Disease, Gladstone Institutes, San Francisco, CA USA; 2 Gladstone Center for Translational Advancement, Gladstone Institutes, San Francisco, CA USA; 3 Biomedical Sciences Program, University of California, San Francisco, San Francisco, CA USA; 4 Neuroscience Program, University of California, San Francisco, San Francisco, CA USA; 5 Developmental and Stem Cell Biology Program, University of California, San Francisco, San Francisco, CA USA; 6 Department of Neurology, University of California, San Francisco, San Francisco, CA USA; 7 Department of Pathology, University of California, San Francisco, San Francisco, CA USA

**Keywords:** Alzheimer's disease, Alzheimer's disease

## Abstract

The full impact of *APOE4* (apolipoprotein E4), the strongest genetic risk factor for Alzheimer’s disease (AD), on neuronal and network function remains unclear, particularly during early preclinical stages of disease. Here we show that young *APOE4* knockin (E4-KI) mice exhibit hippocampal region-specific network hyperexcitability that predicts later cognitive deficits. This early phenotype arises from cell-type-specific subpopulations of smaller, hyperexcitable neurons and is eliminated by selective removal of neuronal *APOE4*. With aging, E4-KI mice develop granule cell hyperexcitability, progressive inhibitory dysfunction and excitation–inhibition imbalance in the dentate gyrus. Single-nucleus RNA sequencing with multilevel gene filtering reveals age-dependent and cell-type-specific transcriptional changes and identifies candidate mediators of early neuronal hyperexcitability, including Nell2. Targeted CRISPR interference knockdown of Nell2 rescues abnormal excitability, implicating Nell2 as a contributor to APOE4-driven dysfunction. Together, these findings define molecular and circuit mechanisms linking neuronal APOE4-induced early network impairment to AD pathogenesis with aging.

## Abstract (web summary)

Young, cognitively normal APOE4 mice display hippocampal network hyperactivity that predicts later memory decline. This phenotype is driven by neuronal APOE4 expression, which causes hyperexcitability of hippocampal neurons and can be reversed by targeting Nell2.

## Main

The ε4 allele of *APOE4* is the strongest genetic risk factor for AD and lowers the age of onset of AD in a gene dose-dependent manner^1^. In most clinical studies, *APOE4* carriers account for 60–75% of all AD cases^2^, highlighting the importance of APOE4 in AD pathogenesis. Longitudinal studies in humans demonstrate that the detrimental effect of APOE4 on cognition is age dependent and occurs before typical signs of AD arise^3^, with *APOE4* carriers exhibiting age-related memory decline earlier in life than non-carriers^4^. Furthermore, structural imaging and cognitive studies have shown that healthy *APOE4* carriers under the age of 40 exhibit reduced cortical thickness^5^ as well as gray matter atrophy and worse cognitive performance^6^. Human *APOE4* knockin (E4-KI) mice lacking amyloid beta (Aβ) accumulation display sex-dependent and age-dependent learning and memory deficits^7,8^. Together, available data suggest that APOE4 plays a role in AD not just in its active stages but actually for decades before disease onset, including the preclinical and prodromal stages. However, there is still no clear understanding of how APOE4 expression promotes AD initiation and progression.

Hippocampal hyperactivation has been reported in young presymptomatic familial patients with AD^9^ and in patients with mild cognitive impairment (MCI)^10^. Subclinical seizures and interictal spikes (IISs) are also common in the preclinical and prodromal stages of AD^11,12^ and are associated with an accelerated cognitive decline^11,13^. Multiple transgenic AD mouse models exhibit network hyperexcitability and seizures preceding Aβ plaque formation^14^, and we previously showed that epileptogenesis in APPswe/PSEN1dE9 mice^15^ is driven by Aβ-induced neuronal hyperexcitability^16,17^. Hippocampal IISs in AD mice resemble those recorded in human patients with epilepsy^18^. Importantly, this hallmark epileptiform activity inversely correlates with memory performance^13,18,19^, and studies found that reducing hippocampal hyperexcitability with the antiepileptic drug levetiracetam improved cognition in amnesic patients with MCI^20,21^ as well as in APP/PS1 (ref. ^22^) and other AD mouse models^23,24^. Collectively, convergent human and animal data indicate that network hyperexcitability contributes to AD-related cognitive decline and represents a tractable therapeutic target^25^.

*APOE4* is associated with region-specific hippocampal hyperactivity in young, non-demented carriers^26–33^ as well as in patients with MCI and early AD^34,35^, with the extent of this hyperactivity predicting future memory decline^34^. Additionally, *APOE4* has been linked to subclinical epileptiform activity during stress^36^. *APOE4* carriers face a higher risk of developing epilepsy^37^, often with earlier onset^38^, particularly after traumatic brain injury^39,40^. Furthermore, *APOE4* confers a six-fold greater likelihood of temporal lobe epilepsy (TLE) treatment resistance^41^ and is linked to impaired memory performance in patients with TLE^42,43^. Notably, young E4-KI mice spontaneously develop seizures starting at 5 months of age—a phenomenon not observed in E3-KI or E2-KI animals—with female E4-KI mice exhibiting higher seizure penetrance^44^. This indicates that APOE4-induced pathophysiological changes in network excitability occur prior to the manifestation of observable cognitive deficits^7^. However, despite reports of increased seizure susceptibility^45^ and penetrance in E4-KI mice, few studies have explored the network-level drivers of these pathological phenotypes. Multiple explanations have been proposed for APOE4-related neuronal hyperactivity^46^ and network dysfunction, including impaired inhibition^46^ and interneuron loss^47^. However, the precise neuronal mechanisms underlying APOE4-induced network hyperexcitability remain poorly understood. Uncovering the causes of APOE4-driven early network pathophysiology is essential to advancing understanding of its contribution to AD pathogenesis.

In the present study, we set out to investigate the mechanisms and consequences of APOE isoform-, age- and region-dependent hippocampal network hyperexcitability. To this end, we employed a multilevel approach combining in vivo local field potential (LFP) recordings from freely moving mice^8^, whole-cell patch-clamp electrophysiology in ex vivo hippocampal slices and single-nucleus RNA sequencing (snRNA-seq) to identify genes that may underlie the observed neuronal functional phenotypes across different ages. Finally, we used targeted CRISPR interference (CRISPRi) to validate key candidate genes as mediators of APOE4-induced selective hippocampal hyperexcitability.

## Results

### Region-specific hippocampal network hyperexcitability in E4-KI mice starting from young ages

**Fig. 1:**
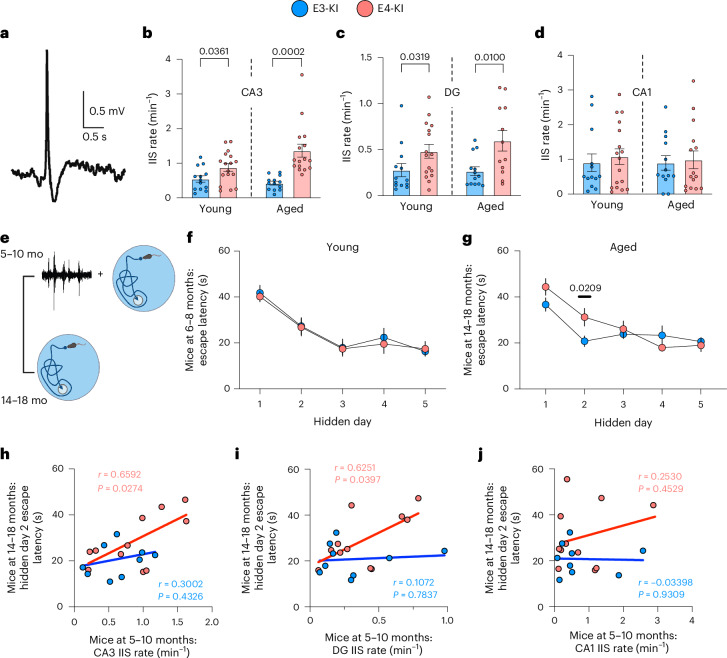
APOE4 induces early region-specific hippocampal network hyperexcitability that predicts future learning impairment. **a**, Example raw LFP trace of an IIS from CA3 PC layer. **b**, CA3 IIS rates in two independent cohorts of young (5–10 months) and aged (12–18 months) E3-KI and E4-KI mice. **c**, IIS rates in the DG of young and aged E3-KI and E4-KI mice. **d**, IIS rates in CA1 of young and aged E3-KI and E4-KI mice. **e**, Timeline of experiments for the longitudinal cohort shown in **f**–**j**. **f**, Average daily escape latency on MWM for the longitudinal E3-KI and E4-KI cohort at 6–8 months of age. **g**, Average daily escape latency on MWM for the longitudinal E3-KI and E4-KI cohort at 14–18 months of age. **h**, CA3 IIS rate at 5–10 months predicts escape latency on hidden platform day 2 trial at 14–18 months in E4-KI mice but not in E3-KI mice. Pearson’s correlation analysis (two-sided). **i**, DG IIS rate at 5–10 months predicts escape latency on hidden platform day 2 trial at 14–18 months in E4-KI mice but not in E3-KI mice. **j**, CA1 IIS rate at 5–10 months does not predict escape latency on hidden platform day 2 trial at 14–18 months in E-KI mice of either genotype. All data are represented as mean ± s.e.m. Significance was assessed using unpaired two-tailed *t*-test or Mann–Whitney *U*-test. For IIS rates (**b**–**d**): *n* = 13 and 17 for young E3-KI and E4-KI mice (16 E4-KI mice for DG IIS); *n* = 13 and 16 for aged E3-KI and E4-KI mice. For MWM tests (**f**,**g**): *n* = 13 each for young E3-KI and E4-KI mice; *n* = 13 and 15 for aged E3-KI and E4-KI mice. For **h**–**j**: *n* = 9 and 11 for E3-KI and E4-KI mice, respectively. mo, months. Icons in **e** created in BioRender; Zilberter, M. https://BioRender.com/tyaw8fo (2025). Source data

Although neuronal hyperactivity was previously reported in the entorhinal cortex of aged E4-KI mice^46^, there is a lack of data on age-dependent APOE4 effects on network excitability in the hippocampus—a brain region critical for spatial learning and memory that is affected early in AD^48^. To investigate age- and APOE4-dependent network activity changes, we analyzed in vivo LFP recordings from two independent cohorts of young (5–10 months) and aged (12–18 months) E-KI mice^8^. We focused on IIS (Fig. 1a), a key biomarker of network hyperexcitability characterized by spontaneous, high-amplitude and brief synchronous discharges of neuronal populations^49^, commonly observed in patients with AD^11^ and in most AD mouse models^14^. We assessed IIS rates across hippocampal cell layers in dorsal CA1, CA3 and dentate gyrus (DG) regions during periods of activity. Our results identified early, APOE4-induced and region-specific network hyperexcitability: starting at a young age, E4-KI mice displayed elevated IIS rates in CA3 (Fig. 1b) and DG (Fig. 1c) but not in the CA1 region (Fig. 1d), compared to age-matched E3-KI animals.

**Extended Data Fig. 1:**
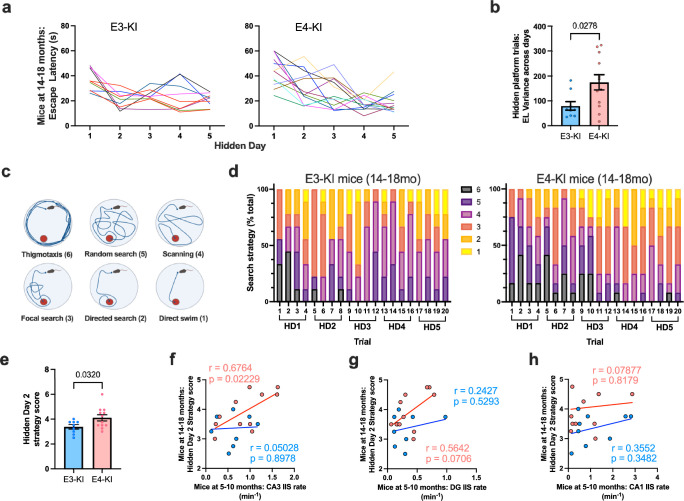
Age-dependent spatial learning and search strategy analysis in E4-KI and E3-KI mice. **a**, Aged E4-KI mice (14-18 months) show significant variability in escape latencies compared to E3-KI mice, reflecting heterogeneity in spatial learning performance. **b**, Average escape latency variance across 5 days of hidden platform trials for each mouse. **c**, Schematic representation of the six distinct search strategies used by mice in the MWM test. **d**, Strategy use as % total for each hidden platform trial across 5 days for aged E3-KI (left) and E4-KI (right) mice. **e**, Average search strategy scores for day 2 of the hidden platform trials. **f**, Early CA3 IIS rates correlate significantly with strategy scores on day 2 of hidden trials in aged E4-KI mice. **g**, Relationships between DG IIS rates in young E-KI mice and 2 of hidden trials strategy scores at old age. **h**, Early CA1 IIS rates do not predict strategy scores on hidden day 2 in aged E-KI mice. All data in (**b, e**) are represented as mean ± s.e.m. For IIS analysis, *n* = 14 and 15 young E3-KI and E4-KI mice; *n* = 13 and 16 for aged E3-KI and E4-KI mice. MWM test, *n* = 9 and 12 for E3-KI and E4-KI mice. Significance was assessed using unpaired two-tailed Student’s t-test or Pearson’s correlation analysis (two-sided). Panel (**c**) created in BioRender https://BioRender.com/6sz2fev. Source data

Despite exhibiting network hyperexcitability, young E4-KI mice did not display spatial learning impairments in the Morris water maze (MWM) test compared to their E3-KI counterparts (Fig. 1f). However, by 14 months of age, the same E4-KI mice developed significant learning deficits, as evidenced by longer escape latencies on day 2 of the hidden platform trials (Fig. 1g), consistent with previous reports of spatial learning decline in aged E4-KI mice^7,8^. To determine whether these increased escape latencies reflected genuine spatial learning impairments rather than non-cognitive factors, we analyzed search strategy patterns used by mice to locate the hidden platform^50^ (Extended Data Fig. 1c). On day 2 of the hidden platform trials, E4-KI mice exhibited a significantly higher reliance on random search strategies, such as repetitive scanning or random searching, resulting in elevated strategy scores (Extended Data Fig. 1d,e). This suggests a failure to learn or recall the platform’s location from previous trials, which would otherwise enable efficient task completion via spatially targeted strategies, such as ‘directed swim’ and ‘focal search’. Thus, the increased escape latencies observed in E4-KI mice appear directly linked to ineffective strategy selection, likely due to impaired spatial learning or recall.

Notably, escape latencies in aged E4-KI mice showed considerable heterogeneity, similar to IIS rates (Extended Data Fig. 1a,b). Leveraging this variability, we examined whether early network hyperexcitability could serve as a predictor of future learning impairments (Fig. 1e). Indeed, IIS rates in the CA3 and DG regions recorded in young E4-KI mice (5–10 months) correlated significantly with escape latencies on hidden day 2 in the same animals at older ages (14–18 months) (Fig. 1h,i), whereas no such correlation was observed in E3-KI mice (Fig. 1h,i) or between CA1 IIS rates and escape latencies in E-KI mice of either genotype (Fig. 1j). Consistent with these findings, CA3 IIS rates in young mice also correlated significantly with day 2 strategy scores in aged E4-KI, but not E3-KI, mice (Extended Data Fig. 1f). The DG IIS rates in young E4-KI mice showed a similar trend that did not reach statistical significance (*r* = 0.56, *P* = 0.071; Extended Data Fig. 1g). Finally, CA1 IIS rates were not predictive of strategy scores in mice of either genotype (Extended Data Fig. 1h). Taken together, these findings indicate that APOE4-induced early hippocampal hyperexcitability, particularly within the CA3 and DG regions, acts as a crucial early marker and potential driver of later cognitive decline.

### APOE4 induces cell-type-specific neuronal hyperexcitability in the hippocampus of young mice

We next investigated the neuronal mechanisms underlying the region-specific hippocampal network hyperexcitability in E4-KI mice. Neuronal intrinsic excitability (IE), which can be measured through several fundamental parameters, was assessed using whole-cell patch-clamp recordings. We focused on rheobase (the minimum depolarizing current required for action potential (AP) initiation), output gain (the relationship between incremental 1-second depolarizing current steps and resulting spiking frequency) and spike latency (the delay to the first AP in response to a 1-second, 800-pA ramped current injection). Additionally, we examined passive neuronal properties that modulate excitability, including input resistance (*R*_in_) and membrane capacitance (*C*_m_), with *C*_m_ being proportional to membrane surface area and reflecting neuronal size^51^.

#### CA3 pyramidal cells

**Fig. 2:**
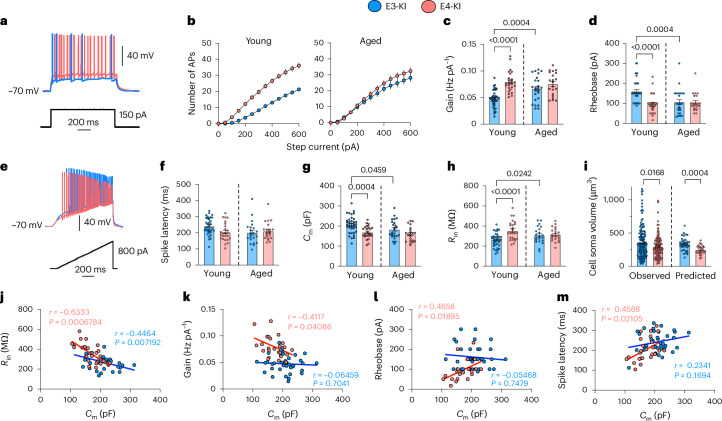
APOE4 drives early intrinsic hyperexcitability and atrophy in CA3 PCs. **a**, Representative whole-cell current-clamp recordings of membrane potential response (top) to a 1-second, 150-pA suprathreshold depolarizing current injection (bottom) in CA3 PCs from young E3-KI and E4-KI mice. **b**, Mean input current–firing rate relationship (I–F curves), the number of spikes elicited by 1-second epochs of incremental 50-pA depolarizing current injection. **c**, Output gain values, quantified as the linear slope of the I–F curve. **d**, Rheobase values, representing the minimum 1-second depolarizing current required to elicit an AP. **e**, Example whole-cell current-clamp recording of spike latency protocol with membrane potential (top) response to a ramped 800-pA, 1-second depolarizing current injection (bottom). **f**, Spike latency values. **g**, Cell *C*_m_ values. **h**, *R*_in_ values. **i**, Cell soma volumes for CA3 PCs from young E3-KI and E4-KI mice, measured directly or predicted using reported specific *C*_m_ of 0.9 pF cm^−^^2^. Statistical differences were analyzed using the Kolmogorov–Smirnov test. **j**, Correlation between *C*_m_ and *R*_in_ values in young E3-KI (blue) and E4-KI (red) CA3 PCs. Numbers represent Pearson’s correlation coefficient and corresponding *P* values. **k**–**m**, Cell *C*_m_ correlates with neuronal excitability in E4-KI but not in E3-KI neurons: correlation between *C*_m_ and output gain (**k**), rheobase (**l**) and spike latency (**m**). Pearson’s correlation analysis (two-sided). All data are represented as mean ± s.e.m. For all panels: young E3-KI: eight mice, *n* = 37 cells (36 in **f**); young E4-KI: seven mice, *n* = 25 cells; aged E3-KI: six mice, *n* = 24 cells; aged E4-KI: four mice, *n* = 21 cells. Significance was assessed using a maximum likelihood ratio test that included age and *APOE* genotype, with *P* values corrected for multiple comparisons using the FDR (5%). Post hoc unpaired two-tailed Student’s *t*-tests or Mann–Whitney *U*-tests were performed between groups if significant genotype or age effects were observed. Source data

**Extended Data Fig. 2:**
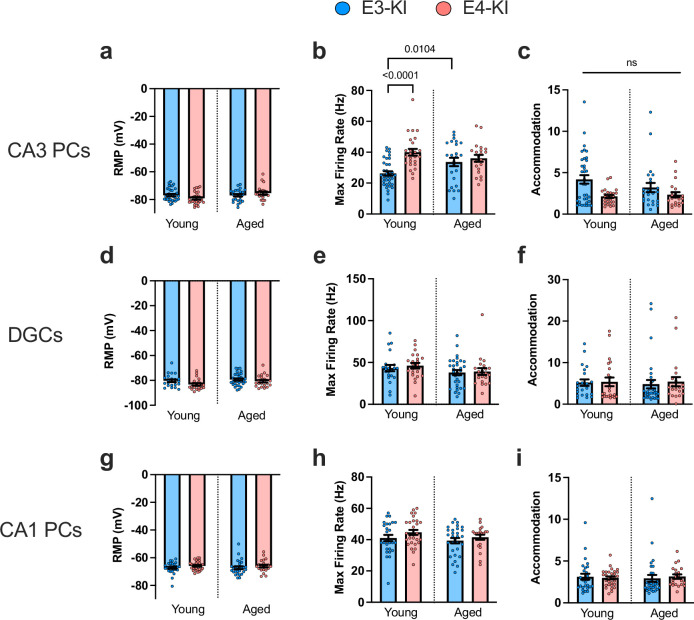
Secondary intrinsic excitability (IE) parameters in hippocampal excitatory neurons from E3-KI and E4-KI mice. **a-c**, CA3 PC IE parameter values: resting membrane potential (RMP) (**a**), maximal firing rate (**b**), and firing accommodation (**c**). **d-f**, Type II DGC IE parameter values: RMP (**d**), maximal firing rate (**e**), and firing accommodation (f). g-i, CA1 PC IE parameter values: RMP (**g**), maximal firing rate (**h**), and firing accommodation (**i**). All data are represented as mean ± s.e.m. CA3 pyramidal cells (**a-c**): Young E3-KI: eight mice, *n* = 36 cells (35 in (**c**)). Young E4-KI: seven mice, *n* = 25 cells. Aged E3-KI: six mice, *n* = 24 cells (23 in (**c**)). Aged E4-KI: four mice, *n* = 21 cells. Dentate granule cells (**d-f**): Young E3-KI: four mice, *n* = 22 cells. Young E4-KI: three mice, *n* = 22 cells (21 in (**d**)). Aged E3-KI: five mice, *n* = 32 cells (31 in (**d**)). Aged E4-KI: five mice, *n* = 21 cells. CA1 pyramidal cells (**g-i**): Young E3-KI: four mice, *n* = 27 cells. Young E4-KI: four mice, *n* = 30 cells. Aged E3-KI: four mice, *n* = 30 cells (28 in (**g**)). Aged E4-KI: six mice, *n* = 21 cells. Significance was assessed using a maximum likelihood ratio test that included age and *APOE* genotype, with p-values corrected for multiple comparisons using the false discovery rate (5%). Post hoc two-tailed Student’s t-tests or Mann-Whitney U tests were performed between groups if significant genotype or age effects were observed. Source data

**Extended Data Fig. 3:**
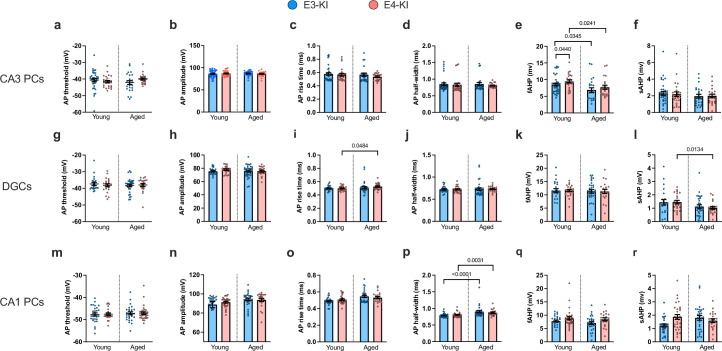
Action potential-associated excitability parameters in hippocampal excitatory neurons from E3-KI and E4-KI mice. **a-f**, CA3 PC action potential (AP) waveform parameters: threshold potential (**a**), amplitude (**b**), rise time (**c**), half-width (**d**), fast afterhyperpolarization, fAHP (**e**), and slow afterhyperpolarization, sAHP (**f**). **g-l**, Type II DGC AP parameters: threshold potential **(g**), amplitude (**h**), rise time (**i**), half-width (**j**), fAHP (**k**), and sAHP (**l**). **m-r**, CA1 PC AP parameters: threshold potential (**m**), amplitude (**n**), rise time (**o**), half-width (**p**), fAHP (**q**), and sAHP (**r**). All data are represented as mean ± s.e.m. CA3 pyramidal cells (**a-f**): Young E3-KI: eight mice, *n* = 36 cells. Young E4-KI: seven mice, *n* = 25 cells. Aged E3-KI: six mice, *n* = 24 cells. Aged E4-KI: four mice, *n* = 21 cells. Dentate granule cells (**g-l**): Young E3-KI: four mice, *n* = 22 cells. Young E4-KI: three mice, *n* = 22 cells. Aged E3-KI: five mice, *n* = 32 cells. Aged E4-KI: five mice, *n* = 21 cells. CA1 pyramidal cells (**m-r**): Young E3-KI: four mice, *n* = 27 cells. Young E4-KI: four mice, *n* = 30 cells. Aged E3-KI: four mice, *n* = 30 cells. Aged E4-KI: six mice, *n* = 21 cells. Significance was assessed using a maximum likelihood ratio test for each parameter, with p-values corrected for multiple comparisons using the false discovery rate (5%). Significance was assessed using a maximum likelihood ratio test that included age and *APOE* genotype, with p-values corrected for multiple comparisons using the false discovery rate (5%). Post hoc two-tailed Student’s t-tests or Mann-Whitney U tests were performed between groups if significant genotype or age effects were observed. Source data

In young E4-KI mice (7–9 months), CA3 pyramidal cells (PCs) exhibited pronounced hyperexcitability compared to those in young E3-KI mice. This was evidenced by a higher output gain (Fig. 2a–c) and lower rheobase values (Fig. 2d), although no significant genotype-mediated differences were observed in spike latency (Fig. 2e,f) when analyzed using a maximum likelihood ratio test with age and genotype as factors. Interestingly, CA3 PCs in aged E3-KI mice (17–19 months) displayed a significant increase in excitability (Fig. 2b–d), which was similar to that of aged E4-KI mice, eliminating the excitability differences seen at younger ages. Other IE-related parameters, such as resting membrane potential (RMP), maximal firing rate and spike accommodation, were also analyzed (Extended Data Fig. 2), as were AP-related parameters (Extended Data Fig. 3). Neurons from young E4-KI mice exhibited an elevated maximal firing rate (Fig. 2b and Extended Data Fig. 2b) and increased fast afterhyperpolarization (fAHP) (Extended Data Fig. 3e) compared to those from E3-KI mice, but no differences were observed in AP amplitude, rise time, half-width or slow afterhyperpolarization (sAHP) (Extended Data Fig. 3a–d,f).

CA3 PCs in young E4-KI mice also had smaller *C*_m_ values (Fig. 2g) paralleled by higher *R*_in_ values (Fig. 2h). Because specific *C*_m_ in neurons is constant at 0.9 μF cm^−^^2^ across all cell types^52^, *C*_m_ serves as a direct representation of membrane surface area and, thus, cell size^51^. Direct measurements of CA3 PC soma volume using confocal microscopy confirmed that E4-KI cells were smaller than those in E3-KI mice (Fig. 2i). Furthermore, *C*_m_ and *R*_in_ values were significantly correlated in both E3-KI and E4-KI neurons (Fig. 2j), demonstrating a link between cell size and input resistance^53,54^. In aged E3-KI mice, CA3 PCs became smaller, as evidenced by lower *C*_m_ and higher *R*_in_ values compared to young E3-KI mice (Fig. 2g,h). The similarity between age-related and genotype-related changes in cell size and IE suggests a causal relationship, as reported in other AD models^55,56^. Indeed, all CA3 PC IE parameters were significantly correlated with *C*_m_ in young E4-KI, but not E3-KI, mice (Fig. 2k–m), indicating that APOE4-induced hyperexcitability is at least partially driven by reduced cell size.

#### Dentate granule cells

**Fig. 3:**
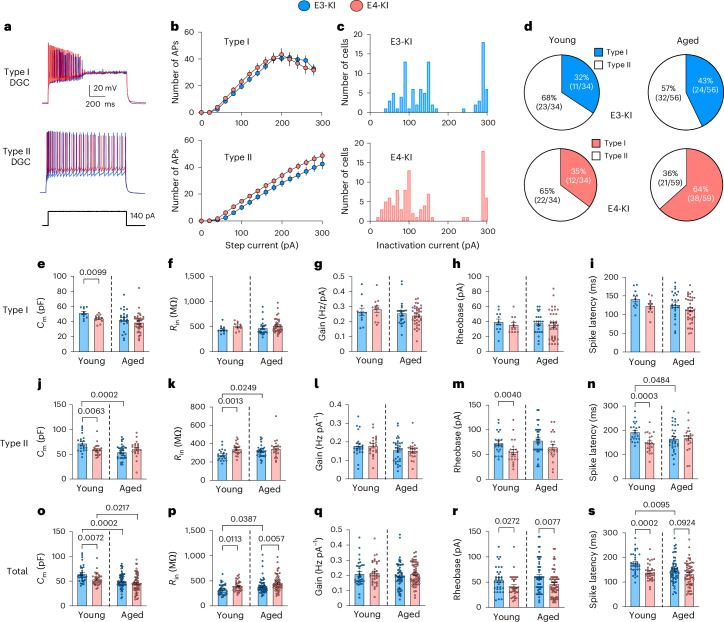
DGC subpopulations display age-specific, *APOE*-genotype-specific and cell-subtype-specific morpho-electric and excitability differences. **a**, Representative whole-cell current-clamp recordings of membrane potential response to a 1-second, 140-pA suprathreshold depolarizing current injection for Type I (top) and Type II (bottom) DGCs in young E3-KI and E4-KI mice. **b**, Mean input current–firing rate (*I*–*F*) curves in Type I (top) and Type II (bottom) DGCs from young E3-KI and E4-KI mice: the number of spikes elicited by 1-second epochs of incremental 20-pA depolarizing current injections. **c**, Histograms of inactivation threshold current values for E3-KI (blue) and E4-KI (red) DGCs across both ages. **d**, Proportions of Type I DGCs and Type II DGCs in E3-KI (blue) and E4-KI (red) young and aged mice. **e**–**i**, Lack of age- or APOE-related IE phenotype in Type I DGCs: values for *C*_m_ (**e**), *R*_in_ (**f**), output gain (**g**), rheobase (**h**) and spike latency (**i**). **j**–**n**, Age- and APOE-dependent changes in morpho-electric parameters of Type II DGCs: values for *C*_m_ (**j**), *R*_in_ (**k**), output gain (**l**), rheobase (**m**) and spike latency (**n**). **o**–**s**, Age-dependent and APOE-dependent changes in morpho-electric parameters in combined DGC population: values for *C*_m_ (**o**), *R*_in_ (**p**), output gain (**q**), rheobase (**r**) and spike latency (**s**). All data are represented as mean ± s.e.m. For all panels: young E3-KI: four mice, *n* = 11 Type I DGCs and 21 Type II DGCs; young E4-KI: three mice, *n* = 12 Type I DGCs (11 in **f**) and 22 Type II DGCs; aged E3-KI: 5 mice, *n* = 24 Type I DGCs (23 in **f**) and 32 Type II DGCs; aged E4-KI: five mice, *n* = 35 Type I DGCs (37 in **e** and 36 in **f**) and 22 Type II DGCs (21 in **n**). Significance was assessed using a maximum likelihood ratio test that included age and *APOE* genotype, with *P* values corrected for multiple comparisons using the FDR (5%). Post hoc unpaired two-tailed Student’s *t*-tests or Mann–Whitney *U*-tests were performed between groups if significant genotype or age effects were observed. Source data

**Extended Data Fig. 4:**
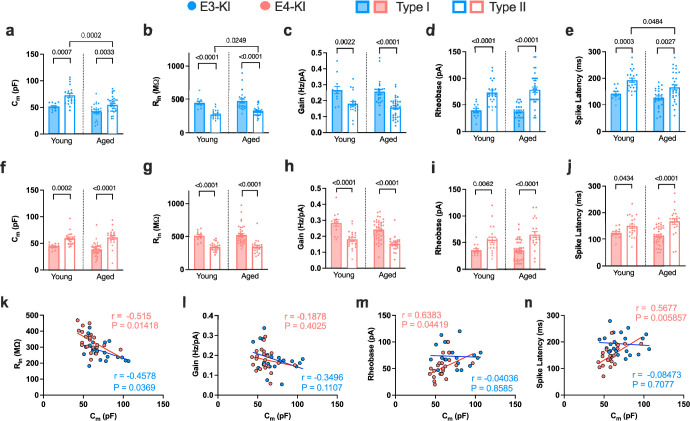
Morpho-electric characteristics defining the two dentate granule cell (DGC) subtypes in E3-KI and E4-KI mice. **a-e**, DGC characteristics for Type I and Type II DGCs in E3-KI mice: *C*_m_ (**a**), *R*_in_ (**b**), output gain (**c**), rheobase (**d**), and spike latency (**e**). **f-j**, DGC characteristics for Type I and Type II DGCs in E4-KI mice: *C*_m_ (**f**), *R*_in_ (**g**), output gain (**h**), rheobase (**i**), and spike latency (**j**). **k**, *C*_m_ and *R*_in_ values correlate in Type II DGC from young E3-KI and E4-KI mice. **l**, *C*_m_ does not correlate with output gain in Type II DGCs (see also Fig. 3l); Pearson’s correlation analysis (two-sided). **m**, *C*_m_ correlates with rheobase in Type II DGCs from E4-KI, but not E3-KI, mice; Pearson’s correlation analysis (two-sided). **n**, *C*_m_ correlates with spike latency in Type II DGCs from E4-KI, but not E3-KI, mice; Pearson’s correlation analysis (two-sided). All data are represented as mean ± s.e.m. For all panels: For all panels: Young E3-KI: four mice, *n* = 11 Type I DGCs, 21 Type II DGCs. Young E4-KI: three mice, *n* = 11 Type I DGCs, 22 Type II DGCs. Aged E3-KI: five mice, *n* = 24 Type I DGCs, 31 Type II DGCs. Aged E4-KI: five mice, *n* = 35 Type I DGCs, 22 Type II DGCs. Significance was assessed using unpaired two-tailed Student’s t-tests or Mann-Whitney U tests. Source data

Among recorded dentate granule cells (DGCs), we identified two subpopulations based on electrophysiological properties. Type I DGCs^57^ were characterized by their inability to sustain firing under higher stimulation intensities (Fig. 3a,b) and exhibited smaller cell size (lower *C*_m_ and higher *R*_in_ values) and greater IE compared to Type II DGCs^57^, regardless of *APOE* genotype or age (Extended Data Fig. 4a–j). Frequency distribution analysis of inactivation thresholds—the depolarizing current step amplitude at which neurons begin to inactivate—revealed two peaks corresponding to Type I and Type II neurons in both *APOE* genotypes (Fig. 3c), indicating distinct cell subpopulations. The proportion of Type I DGCs was similar in young E3-KI and E4-KI mice (Fig. 3d). However, analysis across all groups revealed a significant age–genotype interaction (*P* = 0.0008, Cochran–Mantel–Haenszel test), reflecting an *APOE* genotype-dependent and age-dependent shift in DGC subtype composition, with a higher proportion of Type I DGCs in aged E4-KI mice than in aged E3-KI mice (Fig. 3d).

In young E4-KI mice, the hyperexcitability phenotype was restricted to Type II DGCs, which showed smaller cell size (reduced *C*_m_; Fig. 3j), higher *R*_in_ (Fig. 3k), lower rheobase (Fig. 3m) and shorter spike latency (Fig. 3n), without such effects on Type I DGCs (Fig. 3f,h,i). Correlations between *C*_m_ and IE parameters in E4-KI Type II DGCs further confirmed that reduced cell size contributes to hyperexcitability, similar to observations in CA3 PCs (Extended Data Fig. 4k–n). In aged mice, Type II DGCs showed no significant differences in size or excitability between E3-KI and E4-KI animals, consistent with age-related changes in E3-KI cells (Fig. 3j,k,n). However, the increased proportion of hyperexcitable Type I DGCs (Fig. 3d) sustained the DG hyperexcitability phenotype in aged E4-KI mice (Fig. 3p,r). Finally, no APOE-related differences were observed in secondary IE parameters for DGCs (Extended Data Figs. 2d–f and 3g–l).

#### CA1 PCs

**Extended Data Fig. 5:**
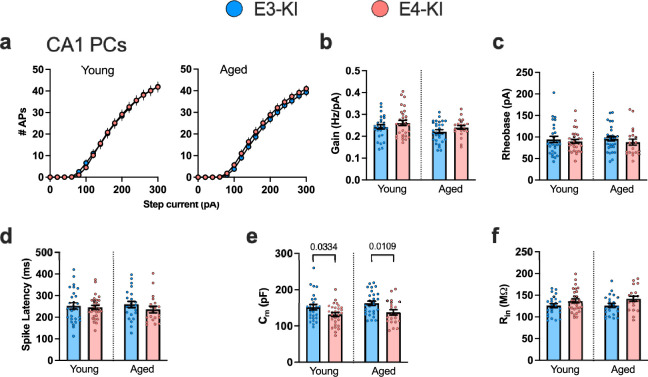
Lack of age- or APOE4-dependent hyperexcitability phenotype in hippocampal CA1 pyramidal cells (PCs). **a**, Input current - firing rate relationship (I-F Curve). **b**, Output gain values. **c**, Rheobase values. **d**, Spike Latency values. **e**, *C*_m_ values. **f**, *R*_in_ values. All data are represented as mean ± s.e.m. For all panels: Young E3-KI: four mice, *n* = 27 cells. Young E4-KI: four mice, *n* = 30 cells. Aged E3-KI: four mice, *n* = 30 cells. Aged E4-KI: six mice, *n* = 21 cells. Significance was assessed using a maximum likelihood ratio test that included age and *APOE* genotype, with p-values corrected for multiple comparisons using the false discovery rate (5%). Post hoc two-tailed Student’s t-tests or Mann-Whitney U tests were performed between groups if significant genotype or age effects were observed. Source data

Consistent with the absence of network excitability differences in the CA1 region (Fig. 1d), we found no significant IE differences in CA1 PCs between E4-KI and E3-KI mice at either age (Extended Data Fig. 5a–d). Although *C*_m_ values were lower in CA1 PCs of E4-KI mice compared to E3-KI at both ages (Extended Data Fig. 5e), the reduction was less pronounced (*C*_m_ in young E4-KI mice: CA3 PCs 82%, Type II DGCs 82%, CA1 PCs 87% versus young E3-KI mice) and did not lead to significant differences in *R*_in_ (Extended Data Fig. 5f). Additionally, secondary excitability parameters of CA1 PCs showed no genotype-dependent differences (Extended Data Figs. 2g–i and 3m–r).

In summary, young E4-KI mice exhibit a cell-type-specific hyperexcitability phenotype in CA3 PCs and DGCs, associated with reduced cell size. By contrast, aged E3-KI mice show similar reductions in neuronal size and increased excitability, likely reflecting normal aging-associated changes^58,59^. These findings indicate that APOE4-induced neuronal hyperexcitability emerges early in life and is driven by neuronal atrophy.

### Removal of APOE4 from neurons rescues the hyperexcitability phenotype in young E4-KI mice

**Fig. 4:**
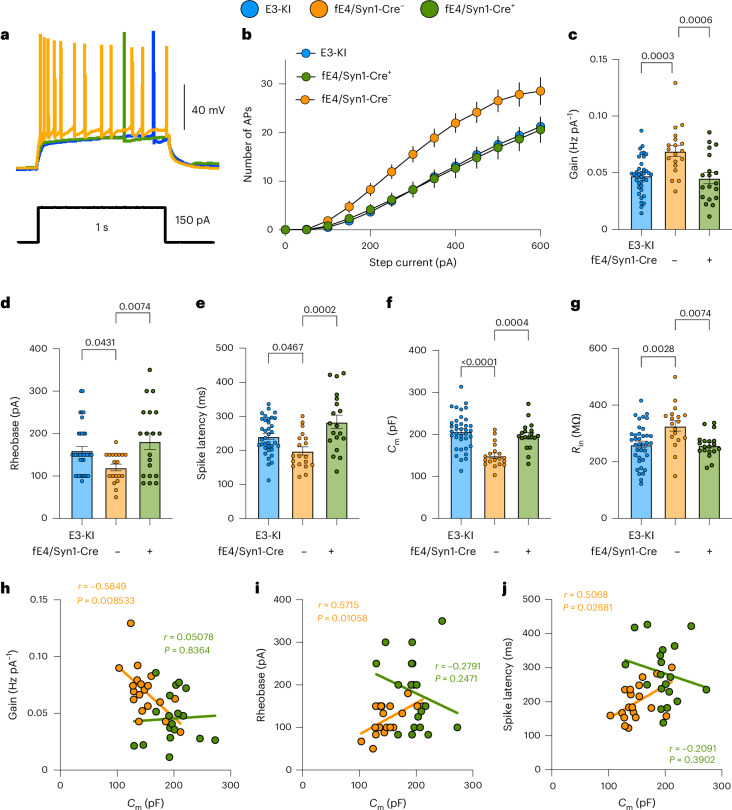
Selective APOE4 removal from neurons rescues morpho-electric and excitability phenotypes of CA3 PCs in young fE4-KI/Syn-Cre^+^ mice. **a**, Representative traces of CA3 PC membrane potential (top) response to a 150-pA, 1-second depolarizing current injection (bottom) in E3-KI, fE4-KI/Syn1-Cre^+^ and fE4-KI/Syn1-Cre^−^ mice. Compared to their Cre^−^ littermates that express *APOE4* in neurons, young fE4-KI/Syn1-Cre^+^ mice display full normalization of all morpho-electric and excitability parameters to E3-KI levels: **b**, input current–firing rate (I–F) curves; **c**, output gain values; **d**, rheobase values; **e**, spike latency values; **f**, *C*_m_ values; **g**, *R*_in_ values. **h**–**j**, Neurons in fE4-KI^Syn1-Cre+^ mice show abolished *C*_m_–IE correlations, whereas neurons in Cre^−^ mice retain these correlations across *C*_m_ versus output gain (**h**), *C*_m_ versus rheobase (**i**) and *C*_m_ versus spike latency (**j**). All data are represented as mean ± s.e.m. All panels: E3-KI: eight mice, *n* = 19 cells; fE4-KI/Syn1-Cre^+^: three mice, *n* = 19 cells; fE4-KI/Syn1-Cre^−^: three mice, *n* = 19 cells. Significance was assessed using one-way ANOVA with post hoc Tukey’s multiple comparisons test. Source data

**Extended Data Fig. 6:**
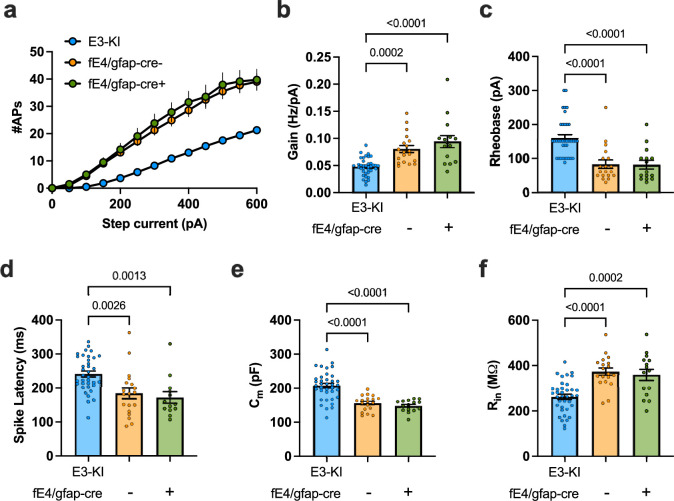
Selective APOE4 removal from astrocytes has no effect on the morpho-electric and excitability phenotypes of CA3 PCs in young fE4-KI^GFAP-Cre+^ mice. **a**, Input current - firing rate relationship (I-F Curves). **b**, Output gain values. **c**, Rheobase values. **d**, Spike Latency values. **e**, *C*_m_ values. **f**, *R*_in_ values. All data are represented as mean ± s.e.m. All panels: E3-KI: eight mice, *n* = 19 cells. fE4-KI^GFAP-Cre+^: three mice, *n* = 15 cells. fE4-KI^GFAP-Cre-^: three mice, *n* = 19 cells. Significance was assessed using one-way ANOVA with Tukey’s post hoc multiple comparisons test. Source data

APOE is primarily made by astrocytes in the brain, although it can also be produced in stressed neurons^60–62^. To determine whether APOE4-related size and excitability phenotypes were driven by neuronal or astrocytic *APOE4* expression^63,64^, we conducted experiments in young E4-KI mice in which the *APOE4* gene was selectively deleted in either neurons (fE4-KI/Syn1-Cre mice^63^) or astrocytes (fE4-KI/GFAP-Cre mice^63^). Neuronal *APOE4* removal fully restored all altered morpho-electric parameters—including *C*_m_ and *R*_in_, output gain, rheobase and spike latency—to the levels similar to those in E3-KI mice (Fig. 4a–g). This emphasizes the critical role of neuronal APOE4 in driving size and excitability changes. Additionally, deleting neuronal *APOE4* abolished all correlations between *C*_m_ and primary IE parameters (Fig. 4h–j), reinforcing the association between neuronal APOE4-induced cell atrophy and hyperexcitability. By contrast, removing astrocytic *APOE4* had no significant effect on electrophysiological properties linked to excitability or cell size (Extended Data Fig. 6). These findings highlight the essential role of neuronal APOE4 in promoting neuronal atrophy and consequent hyperexcitability.

### Selective vulnerability of CA3 PC and DGC subpopulations to APOE4-induced atrophy and hyperexcitability

**Fig. 5:**
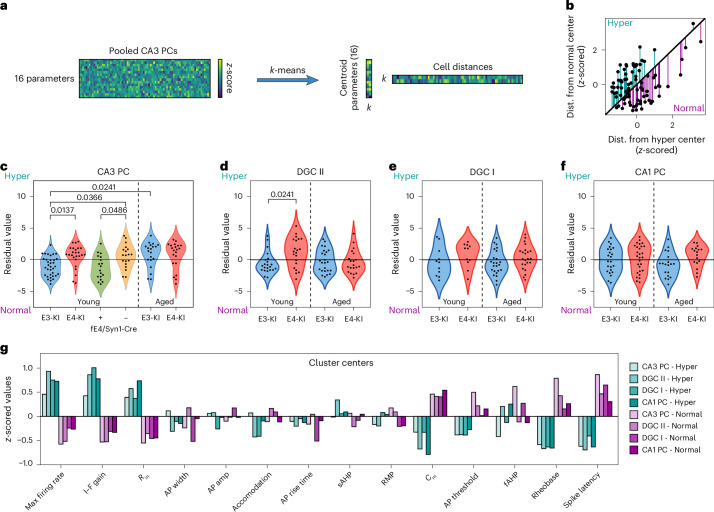
Cluster analysis with *k*-means reveals selective vulnerability of CA3 PCs and DGCs. **a**, Normalized cell parameters for CA3 PCs used for *k*-means clustering analysis. This analysis revealed two primary clusters defined by their centroids and distance to each centroid. **b**, Scatter plot of the distances of each cell to the hyperexcitable or normal cluster centroid. In this plot, the decision line for classification is represented by the unity line. The strength of membership to that cluster can be represented by the residual value of each cell to the unity line. **c**–**f**, Violin plots of the residual values for CA3 PCs (**c**), Type II DGCs (**d**), Type I DGCs (**e**) and CA1 PCs (**f**) across genotypes and age groups. **g**, Centroid loadings for all cell types. For all panels: CA3 PCs: young E3-KI: eight mice, *n* = 34 cells; young E4-KI: seven mice, *n* = 25 cells; aged E3-KI: six mice, *n* = 20 cells; aged E4-KI: four mice, *n* = 21 cells; fE4-KI/Syn1-Cre^+^: three mice, *n* = 18 cells; fE4-KI/Syn1-Cre^−^: three mice, *n* = 19 cells. DGCs: young E3-KI: four mice, *n* = 11 Type I DGCs and 22 Type II DGCs; young E4-KI: three mice, *n* = 11 Type I DGCs and 21 Type II DGCs; aged E3-KI: five mice, *n* = 24 Type I DGCs and 24 Type II DGCs; aged E4-KI: five mice, *n* = 23 Type I DGCs and 20 Type II DGCs. CA1 PCs: young E3-KI: four mice, *n* = 23 cells; young E4-KI: four mice, *n* = 28 cells; aged E3-KI: four mice, *n* = 19 cells; aged E4-KI: four mice, *n* = 17 cells. Significance was assessed using two-way ANOVA with post hoc Tukey’s multiple comparisons test. Source data

**Extended Data Fig. 7:**
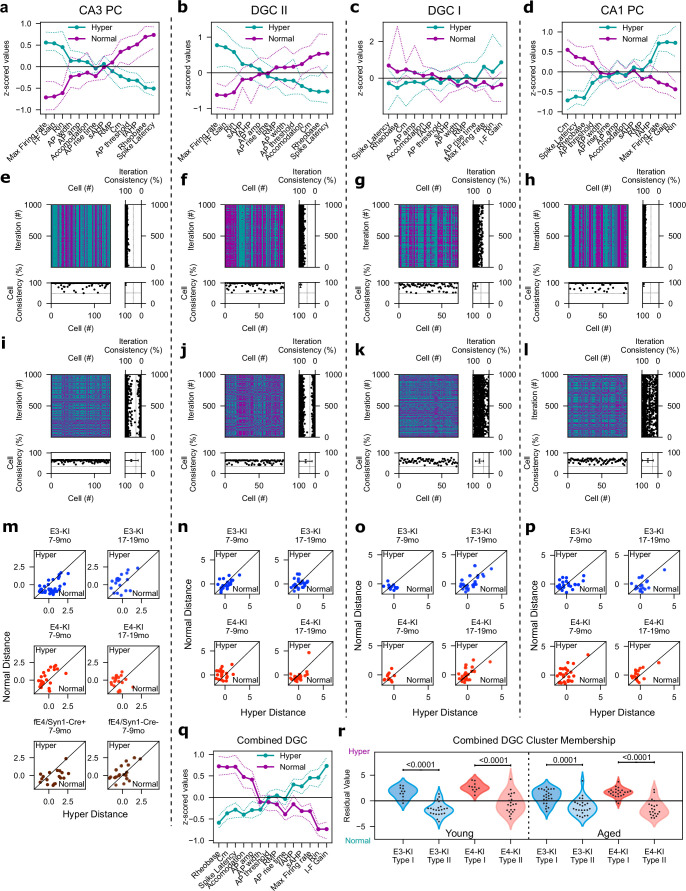
K-means clustering analysis of Type I DGC (DGC I), Type II DGC (DGC II), CA3 PCs, and CA1 PCs. **a-d**, Mean centroids determined by k-means clustering into two clusters for CA3 PCs (**a**), DGC I (**b**), DGC II (**c**) and CA1 PCs (**d**). Dashed lines indicate 99%CI determined from bootstrapping iterations. **e-h**, Consistency analysis for with k = 2 clusters for CA3 PCs (**e**), DGC I (**f**), DGC II (**g**) and CA1 PCs (**h**). For each, heat map shows individual cell classification across each iteration. Upper Right) panel displays the probability of each iteration matching the classification of the full dataset. Bottom Left) panel displays the probability of a cell maintaining is classification identity across iterations. Lower Right) mean probabilities across iterations. Dashed lines indicate chance probability (50%). **i-l**, Consistency analysis for with k = 3 clusters for CA3 PCs (**i**), DGC I (**j**), DGC II (**k**) and CA1 PCs (**l**). **m-p**, Scatter plots indicating the comparative distance from centroid for each cell binned by age and genotype for CA3 PCs (**m**), DGC I (**n**), DGC II (**o**) and CA1 PCs (**p**). Unity line represents the decision line between each hyperexcitable and normal. **q**. Mean centroids from cluster analysis of combined DGCs. **r**) Violin plots of the residual values for combined DGCs across ages, genotype, and heuristically determined DGC types. For all panels: CA3 pyramidal cells: Young E3-KI: eight mice, *n* = 37 cells. Young E4-KI: seven mice, *n* = 25 cells. Aged E3-KI: six mice, *n* = 24 cells. Aged E4-KI: four mice, *n* = 21 cells. fE4-KI^Syn1-Cre+^: three mice, *n* = 19 cells. fE4-KI^Syn1-Cre-^: three mice, *n* = 19 cells. Dentate granule cells: Young E3-KI: four mice, *n* = 11 Type I DGCs, 22 Type II DGCs. Young E4-KI: three mice, *n* = 11 Type I DGCs, 21 Type II DGCs. Aged E3-KI: five mice, *n* = 24 Type I DGCs, 24 Type II DGCs. Aged E4-KI: five mice, *n* = 23 Type I DGCs, 20 Type II DGCs. CA1 pyramidal cells: Young E3-KI: four mice, *n* = 27 cells. Young E4-KI: four mice, *n* = 30 cells. Aged E3-KI: four mice, *n* = 30 cells. Aged E4-KI: four mice, *n* = 21 cells. Significance was assessed two-way ANOVA with Tukey’s post hoc multiple comparisons test. Source data

Significant heterogeneity in neuronal parameters within groups and between genotypes (Figs. 2 and 3) suggests the presence of distinct pathological and healthy subpopulations among CA3 PCs and DGCs. To identify these, we used *k*-means clustering based on morpho-electric properties, pooling neurons across genotypes and ages (Fig. 5a). Clustering revealed two primary groups (*k* = 2), whose robustness was confirmed using a bootstrapping method^65^ (Extended Data Fig. 7a,e). Increasing *k* to 3 did not yield stable clusters (Extended Data Fig. 7i), indicating two dominant subpopulations based on key morpho-electric differences between young E4-KI and E3-KI neurons (Extended Data Fig. 7m). One cluster consistently exhibited hyperexcitable features—lower rheobase, spike latency and *C*_m_, with higher gain and *R*_in_ (Extended Data Fig. 7a)—suggesting that clustering captured key distinctions between hyperexcitable and normal neurons. The covariation of these excitability parameters further supports a hyperexcitable cell state, leading us to heuristically label these clusters as ‘hyperexcitable’ and ‘normal’.

Cells were classified by their distance from cluster centroids, reflecting their similarity to each cluster (Fig. 5a). Scatter plots visualized this process (Fig. 5b), with each neuron’s residual distance to the unity line representing cluster membership strength. *APOE* genotype and age significantly influenced cluster membership, with more hyperexcitable neurons in young E4-KI mice and age-related shifts in E3-KI neurons into the hyperexcitable cluster (Fig. 5c). However, some E4-KI neurons showed a strong association with the normal cluster, indicating selective vulnerability and resilience to APOE4. Deleting neuronal *APOE4* in fE4-KI/Syn1-Cre^+^ mice significantly depleted the hyperexcitable cluster, aligning with E3-KI distribution (Fig. 5c).

Similar analysis in DGCs showed enriched hyperexcitable cluster in Type II DGCs from young E4-KI mice, primarily driven by differences in *R*_in_ and *C*_m_ (Fig. 5d and Extended Data Fig. 7b). Combined DGC population reliably separated into Type I and Type II cells as largely non-overlapping clusters, further confirming their distinct archetypes (Extended Data Fig. 7q,r). In addition, no genotype-related or age-related differences in cluster distributions were seen in Type I DGCs (Fig. 5e) or in CA1 PCs (Fig. 5f).

Overall, centroid comparisons across all cell types revealed consistent divisions, with hyperexcitable cells defined by morpho-electric parameters (*R*_in_ and *C*_m_) and intrinsic excitability (output gain, rheobase and spike latency) but not by AP waveform or other membrane properties (Fig. 5g). Cluster enrichment was driven by cell type, *APOE* genotype and age-related differences in these parameters, confirming that APOE4 expression in specific neuronal subpopulations leads to reduced cell size and hyperexcitability.

### Region-specific and age-dependent synaptic excitation–inhibition imbalance in the hippocampus of E4-KI mice

**Fig. 6:**
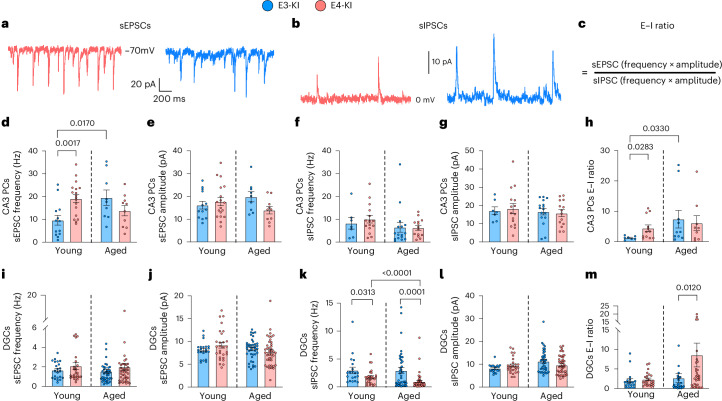
APOE4-driven changes in spontaneous synaptic activity are age dependent and region dependent. **a**, Representative traces of sEPSCs from CA3 PCs in young E4-KI and E3-KI mice. **b**, Representative traces of sIPSCs from DGCs in young E4-KI and E3-KI mice. **c**, Formula for calculating synaptic E–I ratio. **d**, sEPSC frequency in CA3 PCs. **e**, sEPSC amplitudes in CA3 PCs. **f**, sIPSC frequency in CA3 PCs. **g**, sIPSC amplitudes in CA3 PCs. **h**, E–I ratio in CA3 PCs. **i**, sEPSC frequency in DGCs. **j**, sEPSC amplitudes in DGCs. **k**, sIPSC frequency in DGCs. **l**, sIPSC amplitudes in DGCs. **m**, E–I ratio in DGCs. See Extended Data Fig. 8 for cumulative distribution analysis. All data are represented as mean ± s.e.m. CA3 PCs: young E3-KI: five mice, *n* = 13 cells (**d**,**e**) and 7 cells (**f**–**h**); young E4-KI: six mice, *n* = 18 cells (**d**,**e**) and 15 cells (**f**–**h**); aged E3-KI: six mice, *n* = 9 cells (**d**,**e**,**h**) and 16 cells (**f**,**g**); aged E4-KI: five mice, *n* = 10 cells (**d**,**e**,**h**) and 14 cells (**f**–**g**). DGCs: young E3-KI: six mice, *n* = 22 cells; young E4-KI: six mice, *n* = 27 cells; aged E3-KI: seven mice, *n* = 39 cells; aged E4-KI: 10 mice, *n* = 41 cells. Significance was assessed using unpaired two-tailed *t*-tests or Mann–Whitney tests. Source data

**Extended Data Fig. 8:**
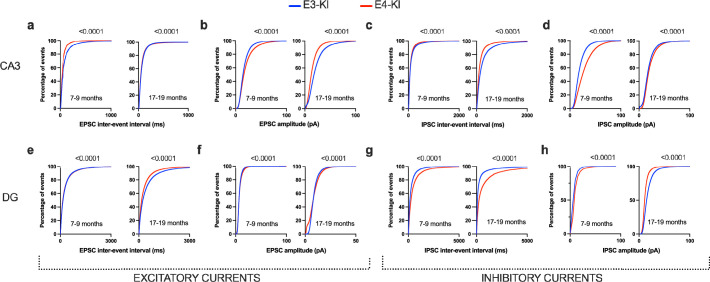
Cumulative distributions for inter-event intervals and amplitudes of all synaptic events shown in Fig. 6. First 1000 events in each recording were used for the analysis. **a**, CA3 PC EPSC inter-event intervals. **b**, CA3 PC EPSC amplitudes. **c**, CA3 PC IPSC inter-event intervals. **d**, CA3 PC IPSC amplitudes. **e**, DGC EPSC inter-event intervals. **f**, DGC EPSC amplitudes. **g**, DGC IPSC inter-event intervals. **h**, DGC IPSC amplitudes. Statistical significance determined using Kolmogorov-Smirnov test. Source data

Another major mechanism contributing to hyperactivity and cognitive deficits in E4-KI mice is synaptic dysfunction, especially the loss of inhibitory function^46,66^. To investigate the network and synaptic consequences of APOE4 expression, we recorded spontaneous excitatory postsynaptic currents (sEPSCs; Fig. 6a) and spontaneous inhibitory postsynaptic currents (sIPSCs; Fig. 6b) in CA3 PCs and DGCs in both young and old age groups. CA3 PCs in young E4-KI mice displayed higher sEPSC frequency and similar amplitude compared to those in young E3-KI mice (Fig. 6d,e) and Extended Data Fig. 8a,b), consistent with hyperexcitability of these cells given the recurrent excitation in CA3 local networks^67^. We found no *APOE* genotype-specific differences in sIPSC frequency or amplitude in CA3 PCs between young E4-KI and E3-KI mice, indicating unaltered inhibitory tone across the *APOE* genotypes at this age (Fig. 6f,g and Extended Data Fig. 8c,d). Increased excitatory drive, together with unchanged inhibitory input, resulted in a higher excitation-to-inhibition (E–I) ratio (Fig. 6c) in CA3 PCs in young E4-KI versus E3-KI mice (Fig. 6h). At old age, both the sEPSC frequency (Fig. 6d) and the E–I ratio (Fig. 6h) significantly increased in CA3 PCs in E3-KI mice, eliminating the differences between *APOE* genotypes and mirroring the age-related excitability changes observed in aged E3-KI neurons.

Finally, we detected no differences in excitatory drive onto E4-KI DGCs at either age (Fig. 6i,j and Extended Data Fig. 8e,f). By contrast, consistent with our prior reports on accelerating hilar interneuron death in E4-KI mice with aging^7,66^, DGCs in E4-KI mice displayed a progressive decline in sIPSC frequency (Fig. 6k and Extended Data Fig. 8g), with no differences in sIPSC amplitude across age or genotype (Fig. 6l), leading to a significantly elevated E–I ratio (Fig. 6m) at old ages.

### Neuronal APOE4 expression leads to neuronal cell-type-specific and age-dependent transcriptomic changes

**Fig. 7:**
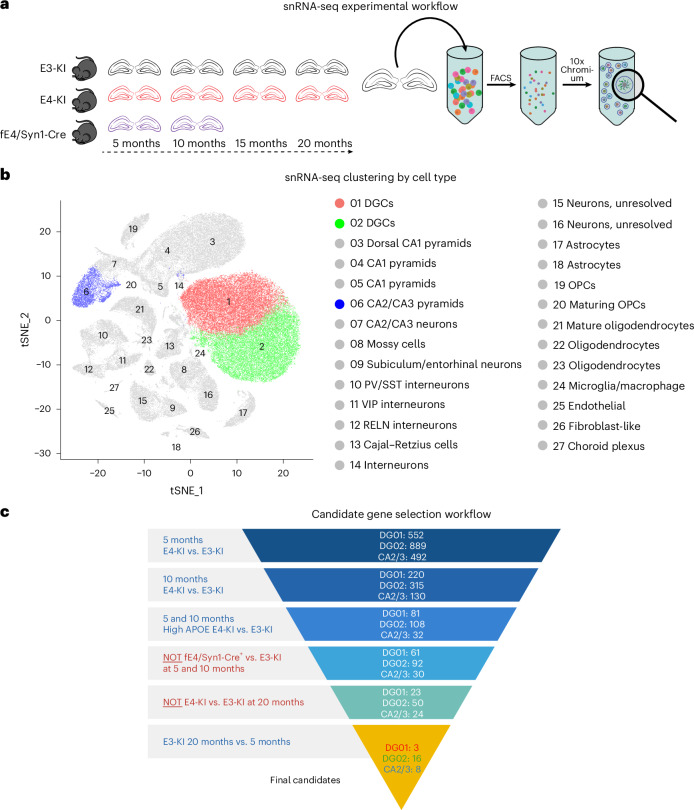
snRNA-seq analysis of E3-KI and E4-KI mouse hippocampi at different ages. **a**, Experimental design: hippocampi were extracted from female E3-KI and E4-KI mice at 5 months, 10 months, 15 months and 20 months of age, and from female fE4-KI/Syn-Cre^+^ mice at 5 months and 10 months of age (*n* = 4 mice per genotype and age). The hippocampi were dissociated, and nuclei were labeled with DAPI and isolated using flow cytometry before processing using the 10x Chromium v2 system for snRNA-seq. **b**, Clustering using the Seurat package revealed 27 distinct cellular populations in E3-KI and E4-KI mice. Marker gene analysis led to the identification of 16 neuronal clusters (clusters 1–16) and 11 non-neuronal clusters (clusters 17–27). **c**, Candidate gene selection criteria for clusters 1, 2 and 6. Values represent the number of genes passing each successive criterion. See also Supplementary Fig. 1 and Supplementary Tables 3–7. OPC, oligodendrocyte progenitor cell; PV, parvalbumin; RELN, reelin; SST, somatostatin; VIP, vasoactive intestinal peptide. Icons in **c** created in BioRender; Zilberter, M. https://BioRender.com/e05iqcb (2025).

The heterogeneous effects of APOE4 across neuronal subtypes resemble the cell-type-specific impact of APOE4 on neuronal transcriptomes that we recently demonstrated^61^. This suggests that a distinct transcriptomic signature may govern the susceptibility or resilience of APOE4-expressing neurons to dysfunction. To uncover APOE4-related gene expression differences in hippocampal neurons, we analyzed our published snRNA-seq datasets obtained from E3-KI and E4-KI mouse hippocampi at different ages (Fig. 7a,b)^61^. To explore the transcriptomic alterations associated with the protective effects of selective *APOE4* deletion in neurons of young E4-KI mice (Fig. 4), we also generated snRNA-seq data from the hippocampi of 5-month-old and 10-month-old female fE4-KI/Syn1-Cre^+^ mice (Supplementary Fig. 1). To select genes of interest whose transcriptional profile changes fit the observed neuronal morpho-electric phenotypes in an *APOE* genotype-, cell type- and age-dependent manner, we focused on clusters 1 and 2 (DGCs) as well as 6 (CA2/CA3 PCs). Because sequencing of the 5-month-old and 10-month-old fE4-KI/Syn1-Cre^+^ was performed independently from our previous study^61^, these data were clustered separately (Supplementary Fig. 1a–f and Supplementary Tables 1 and 2). Integration of the two datasets was not feasible due to significant batch effects, as confirmed by canonical correlation analysis. Therefore, to align cluster identities with those from the original Zalocusky et al. dataset^61^, we calculated the Jaccard similarity index (JSI) using all significant marker genes for each cluster (Supplementary Table 1). Based on JSI analysis, new cluster 1 aligned with original cluster 1, cluster 2 with original cluster 2, cluster 12 with cluster 6 and cluster 5 with cluster 3 (Supplementary Fig. 1g).

For further analysis, we identified genes of interest using six specific criteria (Fig. 7c). We opted to use unadjusted *P* values to reduce stringency and avoid excluding potentially relevant biological candidates because the primary goal of this analysis was to prioritize genes for downstream functional validation. First, genes had to be differentially expressed between E4-KI and E3-KI mice at both 5 months and 10 months of age, as we observed hyperexcitability in E4-KI neurons compared to E3-KI neurons at these timepoints. Second, the genes also had to be differentially expressed in cells with high APOE expression from E4-KI and E3-KI mice at 10 months of age, where cells were classified as ‘APOE-expression-high’ if their APOE mRNA levels were more than 2 s.d. above the median for that cell type. Third, genes had to not be differentially expressed in neurons from fE4KI/Syn1-Cre^+^ mice compared to E3-KI mice at 5 months and 10 months of age, as we found that neuronal *APOE4* deletion resulted in a full normalization of neuronal phenotype. Fourth, genes had to not be differentially expressed in neurons from 20-month-old E4-KI mice compared to 20-month-old E3-KI mice, as no neuronal phenotype was observed in such aged E-KI mice. Lastly, genes had to be differentially expressed in neurons from 20-month-old E3-KI mice compared to 5-month-old E3-KI mice, with expression changes following the same directional pattern as in the earlier criteria, because we observed significant reductions in cell size along with increased excitability in both CA3 PCs and DGCs in aged versus young E3-KI mice (Figs. 2 and 3). From the initial pool of 552 genes for DG cluster 1 (Supplementary Table 3), 889 genes for DG cluster 2 (Supplementary Table 4) and 492 genes for CA2/CA3 PC cluster 6 (Supplementary Table 5) in 5-month-old mice (Fig. 7c), our analysis pipeline yielded three final candidate genes for DG cluster 1 (*Mdga2*, *Shisa4* and *Tnrc6b*), 16 for DG cluster 2 (*Mdga2*, *Nell2*, *Lrrtm4*, *Parvb*, *Plxna4*, *Ubn2*, *Rph3al*, *Adamts17*, *Creld1*, *Cyp7b1*, *Chst9*, *Clta*, *5330417C22Rik*, *Parp8*, *Dpf3* and *Proca1*) and eight for CA2/CA3 PC cluster 6 (*Nell2*, *Dclk1*, *Slit3*, *Ppfia2*, *Lrrc7*, *Nlgn1*, *Rnf182* and *Nnat*) (Supplementary Table 7). By contrast, the same gene analysis on dorsal CA1 PCs (E-KI cluster 3 and young fE4-KI/Syn1-Cre^+^ cluster 5; Supplementary Table 6) yielded no candidates, consistent with these neurons showing no APOE-dependent IE phenotype (Extended Data Fig. 5).

### Targeted reversal of Nell2 overexpression in neurons of young E4-KI mice rescues their smaller size and hyperexcitability phenotypes

**Fig. 8:**
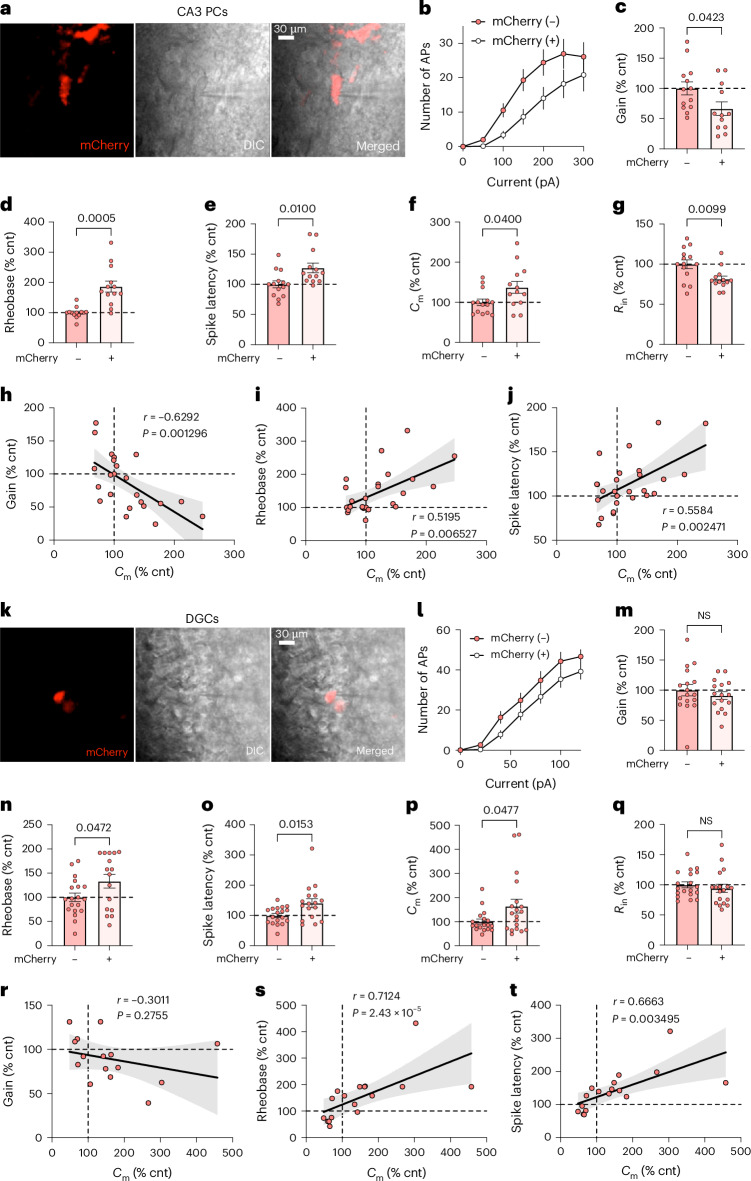
CRISPRi-mediated Nell2 reduction normalizes cell size and excitability of CA3 PCs and DGCs in young E4-KI mice. **a**, Example images of transduced CA3 PCs expressing mCherry (left) targeted for whole-cell patch recording in acute brain slices under differential interference contrast (DIC) imaging (middle). Right: overlay. **c**–**g**, *Nell2* CRISPRi/mCherry^+^ CA3 PCs display normalized morpho-electric and excitability parameters: input current–firing rate (I–F) curves (**b**), output gain (**c**), rheobase (**d**), spike latency (**e**), *C*_m_ (**f**) and *R*_in_ (**g**). **h**–**j**, Increased *C*_m_ correlates with reduced excitability in all recorded CA3 PCs: *C*_m_ versus output gain (**h**), rheobase (**i**) and spike latency (**j**). **k**, Example images of transduced DGCs expressing mCherry (red, left) targeted for whole-cell patch recording in acute brain slices under DIC imaging (middle). Right: overlay. **l**–**t**, *Nell2* CRISPRi/mCherry^+^ DGCs exhibit normalized morpho-electric and excitability phenotypes; no differences were observed in the input–frequency relationship (**l**) or output gain (**m**), consistent with the absence of a phenotype in non-transduced DGCs (Fig. 3b,q). However, *Nell2* CRISPRi led to normalization of rheobase (**n**), spike latency (**o**) and *C*_m_ (**p**), whereas *R*_in_ showed no significant change (**q**). **r**–**t**, Increased *C*_m_ correlates with reduced excitability in all recorded DGCs: *C*_m_ versus output gain (**r**), rheobase (**s**) and spike latency (**t**). Control (‘cnt’) denotes the mean parameter value of mCherry^−^ cells within each mouse; values from that mouse were normalized to this mean and expressed as % cnt. All data are represented as mean ± s.e.m. For CA3 PCs (**b**–**j**): five E4-KI mice, *n* = 13 mCherry^+^ and 12 mCherry^−^ cells. For DGCs (**l**–**t**): four E4-KI mice; *n* = 17 mCherry^+^ cells (16 in **m**, 18 in **q**) and *n* = 19 mCherry^−^ cells (17 in **m**, 18 in **o**,**p**). Significance was assessed using unpaired two-tailed *t*-test or Mann–Whitney *U*-test or Pearson’s correlation analysis (two-sided). NS, not significant. Source data

After identifying several genes that may mediate the detrimental effects of APOE4 on neuronal excitability or modulate susceptibility or resistance to APOE4, we employed the KRAB-mediated CRISPRi gene regulation technique^68^ in young E4-KI mice to assess their functional relevance. Specifically, we aimed to alter their expression toward the levels observed in E3-KI mice. Based on our snRNA-seq results, we selected two candidate genes: *Nell2* (neural epidermal growth factor-like protein 2), the only gene of interest shared between CA2/CA3 PC cluster 6 and DGC cluster 2 (Supplementary Table 7), and *Mdga2* (MAM domain-containing glycosylphosphatidylinositol anchor 2), the only gene of interest shared between DGC clusters 1 and 2 (Supplementary Table 7). To target each selected candidate gene, we used a combination of two custom recombinant lentiviruses: one carrying the gene-specific guide RNA for CRISPRi along with mCherry to fluorescently label transduced neurons and the other carrying a dCas9–KRAB vector. These constructs were injected into the DG/CA3 hippocampal region of 5-month-old female E4-KI mice. Two months later, we conducted patch-clamp recordings to assess electrophysiological parameters in CA3 PCs and DGCs from hippocampal slices of the injected mice. Using mCherry fluorescence as a marker, we were able to distinguish and record from both transduced (CRISPR^+^) and non-transduced (CRISPR^−^) neurons within the same slice (Fig. 8a,k).

**Extended Data Fig. 9:**
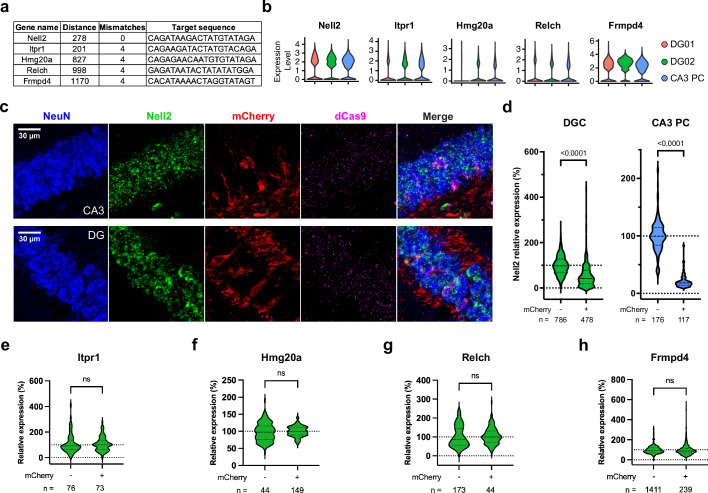
Validation of *Nell2* knockdown by CRISPRi using combined RNAscope and immunohistochemistry. **a**, Details of *Nell2* and estimated CRISPRi off-target genes. **b**, Corresponding gene expression in dentate granule cells and CA3 PCs. **c**, Combined Immunohistochemistry and fluorescent in situ hybridization images CA3 pyramidal cells and dentate granule cells. Neuronal somata are marked with NeuN, transduced cells are marked with mCherry. dCas9 and Nell2 are detected by RNAscope. **d**, Nell2 signal intensity in transduced (mCherry⁺) and non-transduced NeuN⁺/mCherry^−^ cells, normalized to each section’s mCherry^−^ mean. **e**. Itpr1 signal intensity in transduced (mCherry⁺) and non-transduced NeuN⁺/mCherry^−^ cells normalized to each section’s mCherry^−^ mean. **f**. Hmg20a signal intensity in transduced (mCherry⁺) and non-transduced NeuN⁺/mCherry^−^ dentate granule cells normalized to each section’s mCherry^−^ mean. **g**. Relch signal intensity in transduced (mCherry⁺) and non-transduced NeuN⁺/mCherry^−^ dentate granule cells normalized to each section’s mCherry^−^ mean. **h**. Frmpd4 signal intensity in transduced (mCherry⁺) and non-transduced NeuN⁺/mCherry^−^ dentate granule cells normalized to each section’s mCherry^−^ mean. n’s represent the number of cells measured; *n* = 4 E4-KI mice for all experiments. Statistical significance was determined using a linear mixed-effects model with sample identity included as a random effect to account for non-independence of measurements within each section and mouse. Source data

Nell2 is a kinase C-binding glycoprotein^69^ that has been shown to have multiple roles in regulating neuronal proliferation, survival, differentiation and polarization as well as axon guidance and synaptic functions^70–72^. We validated the CRISPRi-mediated reduction of Nell2 expression in mCherry^+^ DGCs and CA3 PCs and confirmed the absence of off-target effects using combined RNAscope and immunohistochemistry (Extended Data Fig. 9). mCherry^+^ *Nell2*-CRISPRi-transduced CA3 PCs and DGCs (Fig. 8a,k) displayed significantly higher rheobase and spike latency values (Fig. 8d,e,n,o) and had higher *C*_m_ values (Fig. 8f,p) compared to neighboring mCherry^−^ non-transduced neurons, indicating reduced excitability and increased size of the *Nell2*-CRISPRi-transduced neurons. mCherry^+^ CA3 PCs also showed decreased output gain (Fig. 8b,c), indicating a rescue of that cell-type-specific phenotype, although we observed no differences in output gain between mCherry^+^ and mCherry^−^ DGCs (Fig. 8l,m), consistent with the lack of APOE4-induced gain phenotype in those neurons (Fig. 3b,l). Notably, changes in *C*_m_ correlated significantly with changes in each of the three phenotypic IE parameters (Fig. 8h–j,s,t), supporting our conclusion that APOE4-induced neuronal atrophy and hyperexcitability phenotypes are related.

**Extended Data Fig. 10:**
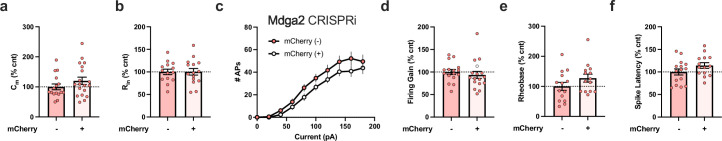
Lack of *Mdga2* CRISPRi effects on neuronal morpho-electric and excitability parameters in young E4-KI mice. *Mdga2* CRISPRi/mCherry+ DGCs display unaltered morpho-electric and excitability parameters: *C*_m_ (**a**), *R*_in_ (**b**), I-F Curves (**c**), output gain (**d**), rheobase (**e**), and spike latency (**f**). Control (‘cnt’) denotes the mean parameter value of mCherry^−^ cells within each mouse; values were normalized to this mean and expressed as % cnt. All data are represented as mean ± s.e.m. For all panels: four E4-KI mice, *n* = 14 mCherry+ and 14 mCherry- cells. Statistical significance determined using unpaired two-tailed t-test or Mann-Whitney U test. Source data

*Mdga2* is a gene previously implicated in autism spectrum disorder (ASD)-related epilepsy^73^. We found no difference in any morpho-electric and excitability parameters in mCherry^+^ *Mdga2*-CRISPRi-transduced neurons versus mCherry^−^ DGCs (Extended Data Fig. 10). Although these data do not support involvement of *Mdga2* in APOE4-induced neuronal morpho-electric phenotype, they do confirm that the CRISPRi procedure itself did not affect neuronal parameters and, therefore, can serve as a ‘negative’ CRISPRi control.

Altogether, our CRISPRi validation study confirms the critical role of Nell2 in mediating early APOE4-induced neuronal morpho-electric abnormalities. These findings demonstrate that targeted reversal of APOE4-driven transcriptomic changes—such as reducing Nell2 expression—may represent a promising strategy for early therapeutic intervention in APOE4-associated AD.

## Discussion

Our findings reveal region-specific hippocampal hyperexcitability in young E4-KI mice, which predicts learning impairment in old age. This pathology is driven by neuronal APOE4 expression, leading to critical transcriptomic changes, including increased expression of Nell2, which promotes neuronal hyperexcitability. The resulting network hyperactivity is further exacerbated by age-related deficits in hilar inhibitory signaling and an imbalance in the E–I ratio, collectively contributing to APOE4-driven pathophysiology and cognitive decline. Notably, early hippocampal hyperexcitability in cognitively normal young E4-KI mice strongly correlates with later spatial learning impairments, aligning with human studies linking hippocampal hyperactivity in young *APOE4* carriers to future cognitive decline^34^. This suggests that early-onset network hyperexcitability is not merely transient but represents a persistent phenomenon and a major risk factor for the initiation and progression of AD in *APOE4* carriers. Supporting this, our previous work demonstrated that reducing hippocampal hyperactivity in 9-month-old E4-KI mice through a 6-week treatment with the GABA_A_ receptor potentiator pentobarbital effectively prevented learning and memory deficits at 16 months^74^. These findings underscore the potential of early interventions targeting network hyperactivity to provide long-term protection against APOE4-induced cognitive decline, emphasizing the critical role of early hyperexcitability in driving AD progression.

In mouse amyloidosis models of AD, network hyperactivity arises from the hyperexcitability of excitatory neurons^16,17,75^ as well as by inhibitory deficits^76^ resulting from Aβ toxicity. Several intrinsic excitability parameters have been identified as mediators of the impact of Aβ on neuronal hyperexcitability, including depolarized neuronal RMPs^16,17^, depolarized reversal potential of GABA-mediated inhibitory synaptic currents^17^, increased output gain^77,78^, reduced spike latency^78^ and altered AP width^55,78^ and amplitude^55^. Although APOE4-induced hippocampal hyperactivity in E4-KI mice shares features with amyloidosis models, we observed important mechanistic distinctions. Notably, APOE4-driven hyperexcitability occurred in the absence of changes in RMP or AP waveform, indicating that while neuronal APOE4 and Aβ converge on hippocampal network hyperactivity, they do so through overlapping yet distinct cellular mechanisms.

Neuronal excitability is also mediated by passive neuronal properties, such as cell size. In our study, CA3 PCs and DGCs in young E4-KI mouse hippocampus were consistently smaller than those in age-matched E3-KI mice, as indicated by lower *C*_m_ values and confirmed by morphometric analysis of cell soma volumes. Our results are consistent with previous reports: APOE4 is associated with smaller neuronal size in resected tissue from human patients with epilepsy^79^. Neuronal APOE4 expression in mice results in reduced dendritic morphology^64^ and has been shown to exert a disruptive effect on dendritic integrity in response to excitotoxic injury^80^. Reduced *C*_m_ has also been reported in human induced pluripotent stem cell (iPSC)-derived neurons from patients with AD^55^ as well as in neurons from APP/PS1 mice^56^. Our analysis showing correlation between *C*_m_ and IE parameters in E4-KI neurons suggests that neuronal atrophy promotes hyperexcitability. This relationship between structural atrophy and increased excitability is consistent with computational models^81–83^; for example, progressive dendritic atrophy in CA3 pyramidal neurons due to chronic stress has been shown to increase input resistance and spike output^83^. Similarly, studies in AD models reported that decreased neuronal size underlies hyperexcitability and hyperactivity of CA1 PCs in APP/PS1 mice^56^, with analogous findings in human iPSC-derived neurons carrying PS1 and APP mutations^55^.

By advanced ages, CA3 PCs and Type II DGCs in E3-KI mice ‘catch up’ with those from E4-KI mice, showing similar reductions in size and increased excitability—likely reflecting the effects of normal aging^58,59^—ultimately eliminating the early morpho-electric differences observed between the genotypes. Interestingly, the results from young fE4/Syn1-Cre^+^ mice, where CA3 PCs lacking APOE expression are indistinguishable in size and excitability from those expressing APOE3, support the notion that APOE4 represents a gain of toxic function and accelerated aging rather than APOE3 exerting a neuroprotective effect. The observed age- and APOE isoform-dependent phenotype aligns with our recent finding in E-KI mice^61^ that a subset of neurons upregulates APOE expression, contributing to selective vulnerability to AD-related pathology and degeneration. Notably, high APOE4 expression peaks at 10 months, whereas high APOE3 expression peaks at 15 months. This progression—from an early E4-KI phenotype to delayed age-related changes in E3-KI mice—parallels epidemiological data indicating that APOE4-associated AD risk begins to increase around age 60, peaks around age 70 (ref. ^84^) and converges with that in APOE3 carriers around age 80 (ref. ^85^). These findings further support the hypothesis that APOE4’s detrimental effects accelerate aging, whereas aging-related changes in APOE3 carriers more likely reflect normal aging effects.

Notably, we found a progressive inhibitory deficit in the DG of E4-KI mice, leading to an E–I imbalance driving DG hyperexcitability at advanced ages. This finding aligns with our previous reports of progressive hilar interneuron loss in these animals^7,66^, which correlated with learning deficits^7^. Inhibitory dysfunction provides a mechanistic link between early network hyperexcitability in young E4-KI mice and learning impairments in aged animals. In support of this, we previously demonstrated that arresting network hyperexcitability with a 6-week pentobarbital treatment in 9-month-old E4-KI mice protected hilar DG interneurons from degeneration and prevented learning impairment 7 months later^74^. Network hyperactivity in E4-KI mice can compromise APOE4-expressing interneurons through various mechanisms, including excitotoxicity and metabolic stress^47^. Neuronal APOE4 expression has been shown to result in neuronal loss after excitotoxic challenges^80^, with interneurons being particularly vulnerable due to their higher energy demands^86^ and lower resistance to chronic stress^87^. Our findings support the hypothesis that APOE4-induced network hyperexcitability in young E4-KI mice drives interneuron loss, promoting network dysfunction and learning deficits in aged animals. Thus, APOE4-induced inhibitory dysfunction likely serves as a critical link between early hippocampal hyperexcitability and cognitive impairment observed in older ages.

One notable observation from our data is that, despite increased excitability of excitatory neurons, aged E3-KI mice do not develop network hyperexcitability or cognitive impairment, consistent with intact compensatory mechanisms—such as preserved inhibitory function—that maintain network homeostasis. By contrast, failure of these mechanisms in E4-KI mice, together with a selective enrichment of smaller, hyperexcitable Type I DGCs, contributes to persistent DG hyperexcitability and age-related learning deficits.

Through snRNA-seq analysis, we identified increased expression of Nell2 in CA3 PCs and DGCs of young E4-KI mice, which was abolished upon the deletion of neuronal APOE4. CRISPRi validation confirmed the critical role of Nell2 in mediating APOE4-induced early neuronal morpho-electric deficits: reducing Nell2 in neurons rescued neuronal size and hyperexcitability phenotype in both CA3 PCs and DGCs of young E4-KI mice. Nell2 is a glycoprotein predominantly expressed by glutamatergic neurons in pyramidal and granule cell layers in CA1–3 and DG, respectively^72^. It has been shown to interact with protein kinase C through its EGF-like domain^69^ and to modulate MAPK activity^70^. Additionally, Nell2 shares domains with thrombospondin-1, a protein involved in regulating cell proliferation and apoptosis^88^. Although Nell2 is secreted as a trophic factor^70,71^, its alternative splicing variant is cytoplasmic and has been implicated in intracellular signaling^89^, including binding to PKCβ1 (ref. ^69^). Our results suggest a cell-autonomous role for the cytoplasmic variant of Nell2 in mediating APOE4 effects, as reducing Nell2 expression in CRISPRi-transduced neurons lowered their excitability, whereas neighboring non-transduced neurons remained hyperexcitable. *NELL2* is part of the ‘neuron cellular homeostasis’ Gene Ontology pathway (GO:0070050), alongside several known AD-associated genes, such as *APP*, *PSEN1*, *PSEN2*, *TMEM106B*, *SLC24A2* and *IL6*. Increased Nell2 protein expression has been observed in the prefrontal cortex of patients with AD across three independent cohorts^90,91^, where it negatively correlates with cognitive function and positively correlates with amyloid levels^92^; elevated Nell2 levels have also been reported in the cerebrospinal fluid (CSF) of patients with AD^92^. These increases were further replicated in AD patient iPSC-derived hippocampal neurons^93^ and in the hippocampus of 5×FAD AD model mice^94^. A recent study analyzing CSF biomarkers in cognitively unimpaired individuals over the age of 65 identified Nell2 as one of five proteins whose CSF levels significantly associated with both total tau and phosphorylated tau (p-tau) levels^95^. Moreover, NELL2 circular RNA (circRNA) expression was found to be upregulated in cortical tissue of patients with AD^96^. Clearly, the role of NELL2 in neuronal morphology and excitability, particularly in the context of APOE4, warrants further in-depth investigation.

Our findings suggest a causative timeline of APOE4-induced network dysfunction with aging. In early stages, neuronal APOE4 expression triggers cell-type-specific transcriptomic alterations, neuronal atrophy and hyperexcitability, culminating in network hyperactivity. As the E4-KI mice age, this process contributes to GABAergic interneuron loss and inhibitory deficits, alongside a pathological increase in hyperexcitable Type I DGCs, ultimately leading to network failure and cognitive impairment. Developing effective interventions for APOE4-related AD will require further research into the precise mechanisms driving APOE4-induced, cell-type-dependent and age-dependent changes in hippocampal excitatory and inhibitory neurons. A deeper understanding of these processes could pave the way for targeted therapeutic approaches to mitigate APOE4-associated pathologies in AD.

## Methods

### Mice

Human E3-KI and E4-KI mice were from Taconic Biosciences. *loxP*-flanked *APOE* knock-in (fE-KI) mice with a conditional deletion of the human *APOE4* gene in select cell types were created as described previously^63^: homozygous fE4-KI (floxed *APOE4*) mice were crossbred with *Gfap*-Cre transgenic mice (6.Cg-Tg(*Gfap-cre*)8Gtm) or Synapsin 1-Cre (*Syn1*-Cre) transgenic mice (B6.Cg-Tg(*Syn1*-cre)671Jxm/J). These crosses produced mice that were heterozygous for *APOE4* and positive for *Gfap*-Cre or *Syn1*-Cre. These mice were further crossbred with fE4-KI mice to generate mice that were homozygous for floxed *APOE4* and positive for *Gfap*-Cre (fE4-KI/GFAP-Cre^+^) or *Syn1*-Cre (fE4-KI/Syn1-Cre^+^). Littermates negative for *Gfap*-Cre or *Syn1*-Cre were used as controls. All mice were on a pure C57BL/6 genetic background and were housed in a pathogen-free barrier facility on a 12-hour light cycle at 19–23 °C and 30–70% humidity. Animals were identified by ear punch under brief isoflurane anesthesia and genotyped by polymerase chain reaction (PCR) of a tail clipping. All animals otherwise received no procedures except those reported in this study. All animal experiments were conducted in accordance with the guidelines and regulations of the National Institutes of Health, the University of California and the Gladstone Institutes under protocol AN176773. All protocols and procedures followed the guidelines of the Laboratory Animal Resource Center at the University of California, San Francisco (UCSF) and the ethical approval of the UCSF Institutional Animal Care and Use Committee.

### In vivo electrophysiology

For in vivo network excitability analysis, we analyzed LFP recording data from our previous study^8^. In brief, data were collected from Neuronexus probes (configuration A4x8-400-200-704-CM32), which had four 5-mm shanks spaced 400 mm apart with eight electrode sites per shank and 200-mm spacing between sites, inserted into dorsal hippocampus. Data from all mice were collected, amplified, multiplexed, processed and digitized using a 32-channel Upright Headstage, Commutator and Main Control Unit (SpikeGadgets). Simultaneous data acquisition at 30 kHz and video tracking at 30 frames per second were conducted with Trodes software (SpikeGadgets). Each data collection period consisted of 5 days of 60-minute home cage sessions. To control for the effects of circadian rhythm, each mouse was recorded at a randomly assigned time each day across the light cycle. The anatomical location of each electrode site was determined by examining Nissl-stained histological sections and raw LFP traces. Data were downsampled to 5 kHz, high-pass filtered at 0.1 Hz and Gaussian smoothed at 2 ms. To remove movement artifacts, all recordings were secondary referenced to a channel located in corpus collosum^97^. LFP recordings were then analyzed using custom software written in MATLAB (MathWorks), incorporating the Chronux (http://chronux.org), Trodes to MATLAB (SpikeGadgets) and NeuroQuery libraries as well as Igor Pro 8 (WaveMetrics) with custom macros. IISs were detected in cell layers during periods of mouse activity as any event exceeding 5 s.d. above baseline for 10–100 ms. The experimenters were blinded to genotypes during surgery and recordings.

### Behavioral tests

For learning performance evaluation, we reanalyzed MWM data from a longitudinal cohort of aged E3-KI and E4-KI mice in our previous study^8^. In brief, mice were kept in the testing room with the arena hidden by a partition and given 2 days to acclimate to the environment in a new cage before starting the training. In each trial, mice were placed in a 122-cm-diameter pool filled with opaque water and had to find a 14 × 14-cm platform submerged 1.5 cm below the water’s surface. The mice could rely only on spatial cues on the walls around the pool to guide their search. During the initial 2 days of pretraining, mice swam down a rectangular channel to locate the platform or were guided there by the experimenter after 90 seconds. After these sessions, the rectangular guides were removed, and the platform was moved to a new location. Over the next 5 days, mice underwent four daily hidden platform trials, where they were released from random locations and given 60 seconds to find the platform. These daily trials were divided into two pairs 10 minutes apart, with a 4-hour break between the pairs. Mice were not randomized or stratified. No mice were excluded from the analysis. The experimenters were blinded to genotypes.

### CRISPRi vector design

The dCas9–KRAB-mediated CRISPRi vectors for *Nell2* and *Mdga2* were custom designed, produced and packaged into lentivirus by VectorBuilder using previously published methods^68,98^. Specifically, multiple CRISPRi single guide RNA (sgRNA) sequences were designed for each target gene using VectorBuilder’s algorithm. For each target, sgRNAs were chosen based on their off-target potential, proximity to the transcript start area and sequence characteristics (excluding sequences with consecutive Cs and Gs). Specifically, the sequence for *Nell2* guide RNA (gRNA) was CAGATAAGACTATGTATAGA, whereas the *Mdga2* gRNA sequence was TACCCTGGTGAGCAGAGCCC. To assess any potential off-target effects of the *Nell2* CRISPRi construct, we used the online tool CCTop^99^ to predict genomic loci with sequence similarity to the target site. Because CRISPRi exerts its strongest regulatory effects in close proximity to transcription start sites (TSSs), particularly within −50 bp to 300 bp of the TSS, we focused our analysis on predicted off-target sites located within 1,200 bp of a potential PAM sequence. We then examined the expression patterns of the corresponding genes in our snRNA-seq dataset and excluded genes not expressed in neurons of interest (CA3 PCs and DGCs). This filtering strategy yielded a list of four off-target gene candidates (Extended Data Fig. 10a,b). Each of the four off-target genes was then analyzed for gene expression in transduced versus non-transduced neurons using RNAscope and confirmed to not be affected by the CRISPRi modification (Extended Data Fig. 9e–h).

For specifics and detailed CRISPRi vector design, see https://en.vectorbuilder.com/vector/VB220401-1008gcz.html (*Nell2*) and https://en.vectorbuilder.com/vector/VB220401-1007wjp.html (*Mdga2*).

### Viral injections

For viral transduction, 5-month-old female E-KI mice were anesthetized with an initial intraperitoneal injection of ketamine/xylazine mix and maintained with 1% isoflurane oxygen mix at a flow rate of 1 l min^−1^. The scalp area was cleaned and hair chemically removed. A small incision in the scalp was made to reveal the midline suture from bregma to lambda. Using a stereotactic frame equipped with a small high-speed drill, 0.5-mm burr holes were drilled through the skull at coordinates 2.1 mm posterior to bregma and ±1.5 mm lateral to the midline, at a depth to reveal the surface of the cortex. A 28-gauge canula attached to a syringe pump was lowered to a depth of 1.5 mm below the cortical surface, and 2 μl of lentivirus was injected at a rate of 500 nl min^−1^. After each delivery, we waited an additional 2 minutes for pressure equalization before the canula was slowly removed. The scalp was sutured closed with silk thread. At the end of surgery, mice were treated with subcutaneous ketofen injection near the incision site and intraperitoneal injections of buprenorphine and one of saline for hydration. Mice were allowed to recover in new cages warmed by a heating pad. At the end of the 4-hour recovery, a second injection of buprenorphine was administered, and cages were returned to primary housing racks.

### Tissue slice preparation

Mice were deeply anesthetized with isoflurane and decapitated. The brain was rapidly removed from the skull and placed in the 4 °C slicing solution comprising 110 mM choline chloride, 2.5 mM KCl, 1.25 mM NaH_2_PO_4_, 26 mM NaHCO_3_, 2 mM CaCl_2_, 1.3 mM sodium pyruvate, 1 mM L-ascorbic acid and 10 mM dextrose. To optimize slice quality and enable recordings from dorsal hippocampal neurons, 300-μm sagittal sections were prepared using a vibratome (Leica, VT1200 S). After slicing, slices were transferred into a vapor interface holding chamber (Scientific Systems Design, Inc.) aerated with 95% O_2_/5% CO_2_ gas mixture and allowed to recover at 34 °C for 1 hour before recording.

### Immunohistochemistry and in situ hybridization

Brain tissue collection occurred after the mice were administered intraperitoneal injections of avertin (Henry Schein) and transcardially perfused with 0.9% saline for 1 minute. Right hemispheres were drop fixed for 48 hours in 4% paraformaldehyde (Electron Microscopy Sciences) and then placed in 1× PBS (Corning). Brains were then switched to 30% sucrose for 48 hours before mounting onto a freeze sliding microtome (Leica) and sectioning into 30-µm slices. Sections were stored in cryoprotectant solutions at −20 °C (30% ethylene glycol, 30% glycerol and 40% 1× PBS). Sections selected for staining were then rinsed three times with 1× PBS with 0.1% Tween 20 (PBS-T) (MilliporeSigma) and incubated for 10 minutes in boiling antigen retrieval buffer (Tris buffer, pH 7.6; Teknova). Next, sections were again rinsed in 1× PBS-T before being incubated for 1 hour in a blocking solution (5% normal donkey serum (The Jackson Laboratory) and 0.2% Triton-X (MilliporeSigma) in 1× PBS), followed by 1 hour in Mouse-on-Mouse (MOM) Blocking Buffer (one drop of MOM IgG in 4 ml of PBS-T) (Vector Laboratories). After the MOM block, the sections were incubated overnight at 4 °C with the primary antibody, which was diluted to an optimal concentration of 1:500 for anti-NeuN (MilliporeSigma). The next day, sections were rinsed three times with 1× PBS-T and then incubated for 1 hour in secondary antibody (donkey anti-guinea pig 594, 1:1,000; Jackson ImmunoResearch) with DAPI (1:20,000). After this, the sections were washed again in PBS-T, mounted on microscope slides (Thermo Fisher Scientific) and coverslipped using ProLong Gold Mounting Medium (Vector Laboratories). Images were captured using an FV3000 confocal laser scanning microscope (Olympus) at ×40 magnification. To minimize batch-to-batch variation, all samples were stained simultaneously and imaged at the same fluorescence intensity. The experimenters were blinded to genotypes.

Double staining in fluorescence in situ hybridization (FISH) (to detect Nell2 and Nell4 potential off-target genes) and immunohistochemistry were performed on 10-μm tissue sections from paraformaldehyde-fixed, frozen brains using the RNAscope Multiplex Fluorescent Reagent Kit v2 (Advanced Cell Diagnostics), following the manufacturer’s instructions (UM 323100, technical note MK 51-150). C1 and C2 probes targeting the gene of interest and dCas9, respectively, were hybridized, with signal amplified using TSA Vivid 520 and TSA Vivid 650 (Advanced Cell Diagnostics, 1:2,000). Primary antibodies included anti-NeuN (guinea pig; Millipore, ABN90; RRID: AB_11205592; 1:100) and anti-mCherry (rabbit; Abcam, ab167453; RRID: AB_2571870; 1:100). Secondary antibodies included donkey anti-guinea pig conjugated to DyLight 405 (Jackson ImmunoResearch, 706-475-148; RRID: AB_2340470, 1:100) and donkey anti-rabbit conjugated to Alexa Fluor 594 (Abcam, ab150076; RRID: AB_2782993; 1:200). Sections were mounted in ProLong Gold Antifade (Thermo Fisher Scientific) and imaged on an Evident FLUOVIEW FV3000 laser scanning confocal microscope. Images were acquired as *z*-stacks with 1-μm step size and 7–10 steps. Regions of interest (ROIs) were defined from maximum intensity projections based on NeuN and mCherry expression. Target gene and dCas9 signal within these ROIs was quantified using summed intensity projections to ensure complete signal capture given the punctate nature of in situ hybridization labeling. To minimize batch-to-batch variation, all samples were stained simultaneously and imaged at the same fluorescence intensity.

### Cell volume analysis

To measure cell soma volumes from stained sections, we used the Python implementation of Cellpose software^100^.

### Whole-cell patch-clamp electrophysiology

Brain slices were placed into a submerged dual-side recording chamber (Warner Scientific, RC-27D) and perfused at 5 ml min^−1^ on both sides with oxygenated artificial cerebrospinal fluid (ACSF) solution at 34 °C, to optimize oxygen delivery. Recording and holding ACSF solution comprised 124 mM NaCl, 26 mM NaHCO_3_, 10 mM glucose, 1.25 mM NaH_2_PO_4_, 2.5 mM KCl, 1.25 mM MgCl_2_ and 1.5 mM CaCl_2_.

Neurons were imaged using a modified Olympus BXW-51 microscope with a ×60 objective (Scientifica, Inc.). Whole-cell patch-clamp recordings were performed using a Multiclamp 700B amplifier with signals sampled at 10 kHz and digitized using a Digidata 1550B with Axon pCLAMP software (all from Molecular Devices). Cells were allowed to stabilize for a minimum of 3 minutes after obtaining whole-cell patch configuration before recordings commenced. Patch pipettes were filled with potassium-gluconate-based solution containing 122.5 mM K-gluconate, 8 mM KCl, 10 mM HEPES, 2 mM MgCl_2_, 0.2 mM EGTA, 2 mM ATPNa and 0.3 mM GTPNa, pH set to 7.2–7.3 with KOH and with osmolarity of 270–280 mOsm. To block spontaneous synaptic activity, AMPA receptor antagonist 6-cyano-7-nitroquinoxaline-2,3-dione (CNQX, 20 μM), NMDA receptor antagonist ᴅ-2-amino-5-phosphonopentanoic acid (AP-5, 50 μM), and GABA_A_ receptor antagonist Picrotoxin (5 μM) were added to the perfusate.

Spontaneous synaptic activity was recorded in standard ACSF using a Cs^+^-based intracellular pipette solution containing 140 mM CsMeSO_4_, 10 mM HEPES, 2 mM MgCl_2_, 0.6 mM EGTA, 2 mM ATPNa and 0.3 mM GTPNa, set to pH 7.2–7.3 with CsOH and with osmolarity of 270–280 mOsm. sEPSCs were recorded at −70 mV, and sIPCSs were recorded at 0 mV, for a period of 3–5 minutes.

### Whole-cell patch-clamp electrophysiology data analysis

Electrophysiological data analysis was performed in Igor Pro 8 (WaveMetrics). Postsynaptic events were quantified using a template matching algorithm in NeuroMatic v.3c plug-in for Igor Pro (Jason Rothman). Experimenters were blinded to genotypes during the analysis. No data were excluded. See Supplementary Methods for further details.

### *k*-means clustering analysis of neuronal morpho-electric parameters

Cluster analysis was performed using Python (version 3.10.12) scripts. For each cell type, clustering was performed with ages and genotypes combined. Electrophysiological measures were normalized to *z*-scores. The *k*-means clustering algorithm implemented by the Scikit-learn package^101^ was applied to the complete set of normalized data as well as subsets using five-fold cross-validation where we randomly selected 80% of the neurons to use for cluster fitting, with the remaining 20% of the neurons classified based on the resulting clusters. This was repeated for 1,000 iterations, and the cluster centroids along with neuronal cluster assignment were recorded for each iteration. The probability of a cell maintaining its membership in the original cluster (Extended Data Fig. 7e–h, bottom panels) and the probability of each iteration to match the clustering of the full dataset were used to validate consistency of the clustering paradigm and to determine appropriate cluster number (*k* value). To calculate strength of membership residuals, the distances from each centroid determined by the *k*-means algorithm were normalized to *z*-scores, and, for each cell, the difference (residual) in distances was used to estimate the affinity for hyperexcitable or normal clusters. These residuals for different genotypes and ages were compared with two-way ANOVAs and post hoc Tukey tests.

### Single-nuclei preparation for 10x loading

For analysis of transcriptome in various types of cells in the hippocampus from E3-KI and E4-KI mice at different ages, the snRNA-seq dataset from our previous publication^61^ (Gene Expression Omnibus (GEO) accession ID: GSE167497) was used for the analysis in the present study. To isolate single nuclei from 5-month-old and 10-month-old fE-KI/Syn1-Cre^+^ mouse brains, we combined and adapted the 10x Genomics-demonstrated protocol for nuclei isolation from adult mouse brain and the Allen Brain Institute protocol for fluorescence-activated cell sorting (FACS) of single nuclei as follows. Hippocampi were acutely dissected on ice. Dissected hippocampi were placed in 2 ml of Hibernate A/B27/GlutaMAX (HEB) medium in a 5-ml tube. The HEB medium was removed to a 15-ml conical tube and kept on ice. Next, 2 ml of chilled lysis buffer (10 mM Tris-HCl, 10 mM NaCl, 3 mM MgCl_2_ and 0.1% Nonidet P40 Substitute in nuclease-free water) was added to the tissue, and the hippocampi were homogenized by suctioning 10 times through a 21-gauge needle. After homogenization, the tissue was lysed on ice for 15 minutes, swirling 2–3 times during this incubation period. The reserved chilled HEB media were then returned to the lysed tissue solution, and the tissue was further triturated with 5–7 passes through a 1-ml pipette. A 30-µm cell strainer (Miltenyi Biotec; MACS SmartStrainer, 130110-915) was washed with 1 ml of PBS, and the lysed tissue solution was filtered through the strainer to remove debris and clumps. Filtered nuclei were centrifuged at 500*g* for 5 minutes at 4 °C. The supernatant was removed, and nuclei were washed in 1 ml of nuclei wash and resuspension buffer (1× PBS with 1.0% BSA and 0.2 U µl^−1^ RNase inhibitor). Nuclei were again centrifuged at 500*g* for 5 minutes at 4 °C and resuspended in 400 µl of nuclei wash and resuspension buffer. DAPI was added to a final concentration of 0.1 µg ml^−1^, and the nuclei were filtered through a 35-µm cell strainer. DAPI^+^ nuclei were sorted by gating on DAPI^+^ events, excluding debris and doublets, using the BD FACSAria II at the Gladstone Institutes’ Flow Cytometry Core.

### Complementary DNA library preparation and sequencing

Complementary DNA (cDNA) libraries were prepared using the 10x Chromium Single Cell 3′ GEM, Library & Gel Bead Kit according to the manufacturer’s instructions. The 10x Chromium v3 kit was used (10x Genomics, 1000092). Libraries were sequenced on an Illumina NovaSeq 6000 sequencer at the UCSF Center for Advanced Technology Core.

### Preprocessing and clustering of 5-month-old and 10-month-old fE-KI/Syn1-Cre^+^ mouse snRNA-seq samples

Demultiplexed FASTQ files were aligned to a custom reference genome built from mm10-1.2.0, which includes introns. This alignment was conducted using the Cell Ranger version 2.0.1 ‘counts’ function with default parameters, as outlined in the Cell Ranger documentation. Unique molecular identifier (UMI) counts were also determined using the Cell Ranger ‘counts’ function, and count matrices from multiple samples were merged into a single count matrix using Cell Ranger’s ‘aggr’ function with default parameters. Barcodes (potential cells) were filtered based on a UMI count threshold. The filtered UMI count matrices were further processed using Seurat version 2.3.4. Specifically, the data were filtered to include only protein-coding genes. Cells were filtered to include only those with 200–2,400 detected genes, 500–4,500 UMIs and less than 0.25% mitochondrial reads, ensuring data quality. To normalize the gene expression matrices, the Seurat NormalizeData function^102,103^ was employed with a scale factor of 10,000. Clustering was performed using the implementation in Seurat version 2.3.4. This algorithm involved embedding cells in a *k*-nearest neighbor graph based on Euclidean distance in principal component analysis space. The edge weights between cells were refined using Jaccard similarity. The Louvain algorithm was used for clustering. Highly dispersed genes were identified using the Seurat FindVariableGenes function^104,105^. These genes were selected based on an average expression range of 0.25–4 and a minimum dispersion of 0.55. Nearest neighbor distances were computed using up to the first 15 principal components and a resolution of 0.6, leading to the identification of 28 distinct clusters.

### Cell type assignment

The t-distributed stochastic neighbor embedding (t-SNE) data visualization technique was used to examine the presence of clusters in which mouse ages and genotypes were intermingled, without any observable indications of batch effects associated with genotype or age. To confirm that cluster identity was not dependent on experimental variables such as age or genotype, we analyzed the proportion of cells from each group within each cluster. A family of *t*-tests was used to compare proportional representation, with *P* values corrected for multiple comparisons using the false discovery rate (FDR) (5%). No significant enrichment of any experimental group was observed in any cluster, based on either adjusted or raw *P* values (Supplementary Table 2). To identify marker genes for each cluster, the FindAllMarkers function in Seurat^102,103^ was used. This algorithm uses the Wilcoxon rank-sum test to iteratively compare gene expression within a putative cluster against the expression in all other clusters. Marker genes (Supplementary Table 1) were selectively chosen to exhibit positive expression (that is, higher expression in the cluster of interest compared to other clusters), to be detected in at least 10% of cells within the cluster and to demonstrate a 0.25 log_2_ fold higher expression in the cluster of interest relative to other clusters. To determine broad cell classes, including excitatory and inhibitory neurons, astrocytes, oligodendrocytes and oligodendrocyte progenitor cells, marker genes were compared against cell-type-specific markers obtained from previous RNA-seq data on sorted cell types^104^. Moreover, for further subdivision of hippocampal cell types, particularly in the identification of principal cell subsets, marker genes for each cluster were compared against hippocampal cell-type-specific marker genes reported in Hipposeq^105^. In the case of less common non-neuronal cell types, we compared gene expression in our cells with those genes enriched in each hippocampal cell type relative to all other cells in the hippocampus, as described in the DropViz resource^106^. To further corroborate the identity of the clusters, we additionally queried the top marker genes for each cluster against the Allen Brain Institute’s genome-wide atlas of gene expression in the adult mouse brain^107^. To align cell types across datasets, we compared the marker genes of the newly generated clusters with those from the original dataset. Cluster similarity was quantified using the JSI, a metric previously shown to be effective in comparing cluster identities in RNA-seq datasets^108^. We focused our analysis on selected clusters from Zalocusky et al.^61^ (clusters 1, 2, 3 and 6) and applied a greedy matching approach when clusters exhibited high similarity to multiple groups. Cluster size was also considered to optimize pairwise matching. For instance, although DGC clusters generally showed strong cross-dataset similarity, clusters 3 and 4 from the 5-month and 10-month fE-KI/Syn1-Cre samples differed substantially in their proportional representation relative to the original DGC clusters (1 and 2), indicating a potentially poor match.

### Candidate gene identification

To find genes that matched the selection criteria of interest based on the significant effects in our morpho-electric data, a set of differential gene expression tests was done between cell populations of interest. Statistically significant differentially expressed genes were detected using the non-parameteric Wilcoxon rank-sum test, which was implemented using the FindMarkers function in Seurat. Only genes that are detected in a minimum fraction of 0.1 in either cell population were considered. A differential expression level threshold of absolute log fold change = 0.05 was used. Although FDR-adjusted *P* values were calculated for multiple comparisons, raw *P* values were used to identify candidate genes for downstream functional validation. Genes with *P* < 0.05 were considered nominally altered.

### Statistics and reproducibility

No statistical methods were used to predetermine sample sizes, but our sample sizes are similar to those reported in our previous publications^16,17,109,110^. Igor Pro and GraphPad Prism were used for analysis with values in text and figures. Prior to statistical testing, each dataset was assessed for normality using the Shapiro–Wilk test. To examine the significant effects of age and *APOE* genotype on neuronal excitability, we compared a maximum likelihood model that included age and *APOE* genotypes with a null model that disregarded them. The resulting *P* values for each of the excitability parameters were adjusted for multiple comparisons using the FDR method (FDR = 5%). For parameters exhibiting significant age or *APOE* genotype effects, we performed focused comparisons between E3-KI and E4-KI mice, or between two different ages within each *APOE* genotype, using two-tailed, unpaired Student’s *t*-tests (for normally distributed data) or two-tailed, unpaired non-parametric tests (for non-normally distributed data), without applying any additional correction methods. For experiments involving three genotypes (E3-KI, fE4/Cre^−^ and fE4/Cre^+^), statistical significance was assessed using one-way ANOVA followed by Tukey’s post hoc multiple comparisons test. Maximum likelihood test and FDR correction were performed using custom scripts in Python using the Scikit-learn and statsmodels packages. Exact statistical tests used and number of mice and cells (*n*) are specified in the figure legends. Statistical differences were deemed significant with *α* values of *P* < 0.05. All datasets included three or more biological replicates. Animals were assigned to experimental groups based on genotype and age, with littermates distributed across groups when possible. Blinding and data exclusion are described in each technique Methods subsection.

### Reporting summary

Further information on research design is available in the Nature Portfolio Reporting Summary linked to this article.

## Supplementary information

Supplementary information Supplementary Fig. 1 and Methods.

Reporting Summary

Supplementary Tables Supplementary Tables 1–7.

## Source data

Source Data Figs. 1–6 and 8 Source data for all main figures.

Source Data Extended Data Figs. 1–10 Source data for all extended data figures.

## Data Availability

All data associated with this study and the information of used materials are available in the main text, Methods or Supplementary Information. The snRNA-seq datasets of E3-KI and E4-KI mice at different ages are used from our previous publication^61^ (GEO accession ID: GSE167497). The snRNA-seq datasets of fE-KI/Syn1-Cre mice generated during the study are available at the GEO (accession ID: GSE279550). Source data are provided with this paper.

## Code availability

All code generated during this study is accessible upon reasonable request to the corresponding authors. *k*-means clustering code is available as a GitHub repository (https://github.com/dtabuena/TabuenaJangGrone).
