# Supplementary material for: Neuronal APOE4-induced early hippocampal network hyperexcitability in Alzheimer’s disease pathogenesis
Source: Nat Aging. 2026 Apr 3;6(4):886–904. doi: 10.1038/s43587-026-01096-0 (PMC13099648; doi:10.1038/s43587-026-01096-0)
Supplement: Supplementary file 1 — Supplementary Fig. 1 and Methods. [file 43587_2026_1096_MOESM1_ESM.pdf]

nature aging

Supplementary information https://doi.org/10.1038/s43587-026-01096-0

Neuronal APOE4-induced early hippocampal network hyperexcitability in Alzheimer's disease pathogenesis

In the format provided by the authors and unedited

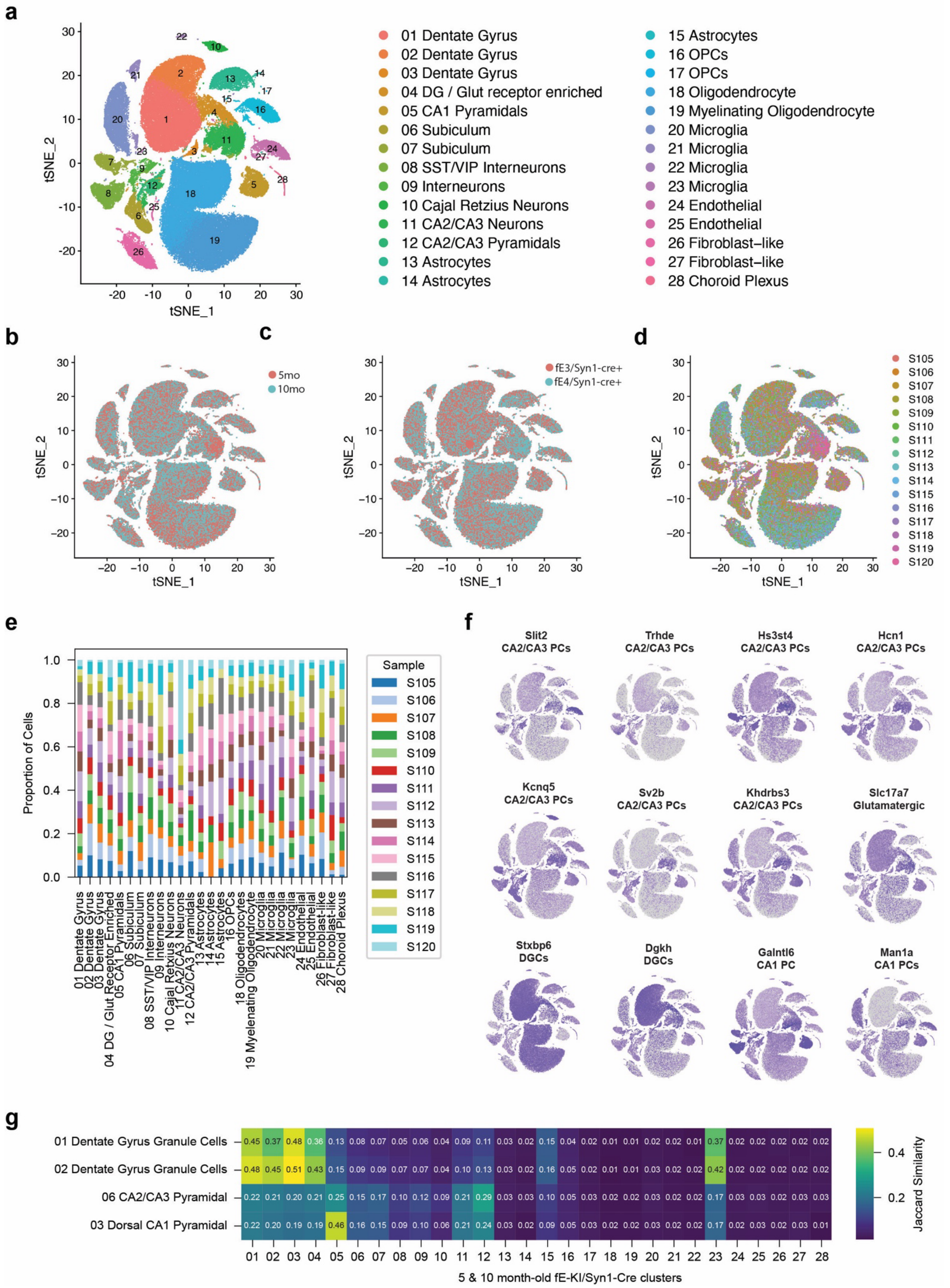
a
tSNE_1
tSNE_2
01 Dentate Gyrus
02 Dentate Gyrus
03 Dentate Gyrus
04 DG / Glut receptor enriched
05 CA1 Pyramidals
06 Subiculum
07 Subiculum
08 SST/VIP Interneurons
09 Interneurons
10 Cajal Retzius Neurons
11 CA2/CA3 Neurons
12 CA2/CA3 Pyramidals
13 Astrocytes
14 Astrocytes
15 Astrocytes
16 OPCs
17 OPCs
18 Oligodendrocyte
19 Myelinating Oligodendrocyte
20 Microglia
21 Microglia
22 Microglia
23 Microglia
24 Endothelial
25 Endothelial
26 Fibroblast–like
27 Fibroblast–like
28 Choroid Plexus
b
5mo
10mo
c
fE3/Syn1-cre+
fE4/Syn1-cre+
d
S105 S106 S107 S108 S109 S110 S111 S112 S113 S114 S115 S116 S117 S118 S119 S120
e
Proportion of Cells
Sample
f
Slit2 CA2/CA3 PCs
Trhde CA2/CA3 PCs
Hs3st4 CA2/CA3 PCs
Hcn1 CA2/CA3 PCs
Kcnq5 CA2/CA3 PCs
Sv2b CA2/CA3 PCs
Khdrbs3 CA2/CA3 PCs
Slc17a7 Glutamatergic
Stxbp6 DGCs
Dgkh DGCs
Galntl6 CA1 PC
Man1a CA1 PCs
g
01 Dentate Gyrus Granule Cells: 0.45 0.37 0.48 0.36 0.13 0.08 0.07 0.05 0.06 0.04 0.09 0.11 0.03 0.02 0.15 0.04 0.02 0.01 0.01 0.02 0.02 0.01 0.37 0.02 0.02 0.02 0.02 0.02
02 Dentate Gyrus Granule Cells: 0.48 0.45 0.51 0.43 0.15 0.09 0.09 0.07 0.07 0.04 0.10 0.13 0.03 0.02 0.16 0.05 0.02 0.01 0.01 0.03 0.02 0.02 0.42 0.02 0.02 0.02 0.02 0.02
06 CA2/CA3 Pyramidal: 0.22 0.21 0.20 0.21 0.25 0.15 0.17 0.10 0.12 0.09 0.21 0.29 0.03 0.03 0.10 0.05 0.03 0.02 0.02 0.03 0.02 0.02 0.17 0.03 0.03 0.02 0.03 0.03
03 Dorsal CA1 Pyramidal: 0.22 0.20 0.19 0.19 0.46 0.16 0.15 0.09 0.10 0.06 0.21 0.24 0.03 0.02 0.09 0.05 0.03 0.02 0.02 0.03 0.02 0.02 0.17 0.02 0.03 0.02 0.03 0.01
01 02 03 04 05 06 07 08 09 10 11 12 13 14 15 16 17 18 19 20 21 22 23 24 25 26 27 28
5 & 10 month-old fE-KI/Syn1-Cre clusters
Jaccard Similarity
0.4
0.2

Supplementary Data Figure 1. Generation of snRNA-seq datasets from the hippocampi of fE-KI$^{Syn1\text{-}Cre+}$ mice for analysis of neuron-specific ApoE effects. Hippocampal snRNA-seq data were collected from fE4$^{Syn1\text{-}Cre+}$ and fE3$^{Syn1\text{-}Cre+}$ mice at 5 and 10 months (N = 4 mice per genotype per age, total 16 mice). The hippocampi were dissociated, and nuclei were labeled with DAPI and isolated using flow cytometry before processing using the 10x Chromium v3 system**.** **a**, The snRNA-seq data were filtered, normalized, and clustered using the Seurat package, which yielded 28 distinct cellular clusters, including 12 neuronal clusters (Clusters 1-12) and 16 non-neuronal clusters (Clusters 13-28). Cell type identities for each cluster were identified using positively enriched marker genes. The tSNE plot shows numbered clusters; the corresponding key shows cell types for each numbered cluster. **b-d**, Single nuclei from both ages (b), genotypes (c) and individual mice (d) are distributed across all 28 clusters. **e**, Proportionality of sample contribution to each cell type cluster. **f,** Feature plots of marker genes for primary excitatory cell types. **g,** Heatmap of Jaccard similarity of marker gene lists between selected clusters from Zalocusky et al. and fE-KI$^{Syn1\text{-}Cre}$ clusters.

SUPPLEMENTARY METHODS

Intrinsic neuronal excitability and passive parameter measurements.

Cell membrane capacitance (C_m). C_m was measured in voltage clamp configuration at -70mV using Clampex software's Membrane Test protocol as the integral of the fast capacitance transient current resulting from a 10mV, 5ms square pulse stimulation.

Input resistance (R_{in}). R_{in} was measured in current clamp configuration at V_m of -70mV using 1s, hyperpolarizing 10pA current injections from -50 pA. Resulting I-ΔV_m curve was fitted with a linear slope to calculate the input resistance.

Resting Membrane Potential was measured in current clamp configuration at I=0.

Input current-spiking frequency (I-F). I-F relationship was recorded in current clamp configuration using incremental 1-s 20pA (CA1 PCs and DGCs) or 50pA (CA3 PCs) depolarizing current injections at 0.1Hz with membrane potential held at -70mV. Resulting spike count was then plotted against the current step magnitude to generate the I-F curve.

Output gain. Output gain was calculated as the linear slope of the I-F curve starting from rheobase.

Rheobase. Rheobase was determined from -70mV as the minimum magnitude of the 1s depolarizing current injection required to elicit an action potential.

Spike latency. Spike latency was measured in current clamp configuration from -70mV using a 1-s 800pA depolarizing current ramped injection, as the delay from the beginning of the stimulation to the generation of the first action potential (when dV_m reached 20V/s).

Accommodation. Accommodation was measured as the ratio of last to first interspike interval in response to a 1-s step current injection at twice the rheobase.

Action potential threshold. Action potential threshold was measured at rheobase step as the V_m value where dV_m crossed 20V/s.

Fast afterhypolarization (fAHP). fAHP was measured as a negative-going peak following the action potential relative to the action potential threshold under a 1s current step injection that elicited at least 4 action potentials.

Slow afterhypolarization (sAHP). sAHP was measured as the negative-going peak relative to baseline within a 1s period immediately after 1s 300pA current step injection.

Spontaneous synaptic activity. Spontaneous excitatory postsynaptic currents (sEPSCs) were recorded at -70mV, inhibitory postsynaptic currents (sIPCSs) at 0mV, for a period of 3-5 minutes.
