# Supplementary material for: Neuronal APOE4-induced early hippocampal network hyperexcitability in Alzheimer’s disease pathogenesis
Source: Nat Aging. 2026 Apr 3;6(4):886–904. doi: 10.1038/s43587-026-01096-0 (PMC13099648; doi:10.1038/s43587-026-01096-0)
Supplement: Supplementary file 2 — Reporting Summary [file 43587_2026_1096_MOESM2_ESM.pdf]

nature portfolio

Corresponding author(s): Misha Zilberter, Yadong Huang

Last updated by author(s): Dec 19, 2025

Reporting Summary

Nature Portfolio wishes to improve the reproducibility of the work that we publish. This form provides structure for consistency and transparency in reporting. For further information on Nature Portfolio policies, see our Editorial Policies and the Editorial Policy Checklist.

Statistics

For all statistical analyses, confirm that the following items are present in the figure legend, table legend, main text, or Methods section.

n/a	Confirmed	
☐	☒	The exact sample size (*n*) for each experimental group/condition, given as a discrete number and unit of measurement
☐	☒	A statement on whether measurements were taken from distinct samples or whether the same sample was measured repeatedly
☐	☒	The statistical test(s) used AND whether they are one- or two-sided<br>*Only common tests should be described solely by name; describe more complex techniques in the Methods section.*
☐	☒	A description of all covariates tested
☐	☒	A description of any assumptions or corrections, such as tests of normality and adjustment for multiple comparisons
☐	☒	A full description of the statistical parameters including central tendency (e.g. means) or other basic estimates (e.g. regression coefficient) AND variation (e.g. standard deviation) or associated estimates of uncertainty (e.g. confidence intervals)
☐	☒	For null hypothesis testing, the test statistic (e.g. *F*, *t*, *r*) with confidence intervals, effect sizes, degrees of freedom and *P* value noted<br>*Give P values as exact values whenever suitable.*
☒	☐	For Bayesian analysis, information on the choice of priors and Markov chain Monte Carlo settings
☒	☐	For hierarchical and complex designs, identification of the appropriate level for tests and full reporting of outcomes
☐	☒	Estimates of effect sizes (e.g. Cohen's *d*, Pearson's *r*), indicating how they were calculated

Our web collection on statistics for biologists contains articles on many of the points above.

Software and code

Policy information about availability of computer code

Data collection	Ex-vivo patch-clamp electrophysiological data was acquired using Pclamp 11 (Molecular Devices). RNAscope, immunohistochemistry, and snRNA-seq data was acquired using drivers for microscopes and sequencing machines.
Data analysis	Analyses were conducted using the following publicly-available software packages: For patch-clamp electrophysiology: Ilgor Pro v8 with NeuroMatic v.3c plug-in, and Python v3.10.12 with Scikit-learn and statsmodels packages. For in-vivo electrophysiology: Igor Pro v8 and MATLAB incorporating the Chronux, Trodes to MATLAB (SpikeGadgets), and Neuroquery libraries. snRNA-seq data analysis was performed using Seurat v2.3.4 and Cell Ranger v2.0.1. RNAscope and neuronal soma size analysis was performed using ImageJ and Python implementation of the Cellpose software. GraphPad Prism v10 was used for statistical analyses and graphing.

For manuscripts utilizing custom algorithms or software that are central to the research but not yet described in published literature, software must be made available to editors and reviewers. We strongly encourage code deposition in a community repository (e.g. GitHub). See the Nature Portfolio guidelines for submitting code & software for further information.

Data

Policy information about availability of data

All manuscripts must include a data availability statement. This statement should provide the following information, where applicable:

- Accession codes, unique identifiers, or web links for publicly available datasets
- A description of any restrictions on data availability
- For clinical datasets or third party data, please ensure that the statement adheres to our policy

All data associated with this study and the information of used materials are available in the main text, the Materials and Methods, or the Supplementary Information section. The snRNA-seq datasets of E3-KI and E4-KI mice at different ages are used from our previous publication9 (GEO accession: GSE167497). The snRNA-seq datasets of fE-KISyn1-cre mice generated during the study are available at GEO (accession: GSE279550). Data associated with all figures are also available as Source Data.

Research involving human participants, their data, or biological material

Policy information about studies with human participants or human data. See also policy information about sex, gender (identity/presentation), and sexual orientation and race, ethnicity and racism.

Reporting on sex and gender	*Use the terms sex (biological attribute) and gender (shaped by social and cultural circumstances) carefully in order to avoid confusing both terms. Indicate if findings apply to only one sex or gender; describe whether sex and gender were considered in study design; whether sex and/or gender was determined based on self-reporting or assigned and methods used. Provide in the source data disaggregated sex and gender data, where this information has been collected, and if consent has been obtained for sharing of individual-level data; provide overall numbers in this Reporting Summary. Please state if this information has not been collected. Report sex- and gender-based analyses where performed, justify reasons for lack of sex- and gender-based analysis.*
Reporting on race, ethnicity, or other socially relevant groupings	*Please specify the socially constructed or socially relevant categorization variable(s) used in your manuscript and explain why they were used. Please note that such variables should not be used as proxies for other socially constructed/relevant variables (for example, race or ethnicity should not be used as a proxy for socioeconomic status). Provide clear definitions of the relevant terms used, how they were provided (by the participants/respondents, the researchers, or third parties), and the method(s) used to classify people into the different categories (e.g. self-report, census or administrative data, social media data, etc.) Please provide details about how you controlled for confounding variables in your analyses.*
Population characteristics	*Describe the covariate-relevant population characteristics of the human research participants (e.g. age, genotypic information, past and current diagnosis and treatment categories). If you filled out the behavioural & social sciences study design questions and have nothing to add here, write "See above."*
Recruitment	*Describe how participants were recruited. Outline any potential self-selection bias or other biases that may be present and how these are likely to impact results.*
Ethics oversight	*Identify the organization(s) that approved the study protocol.*

Note that full information on the approval of the study protocol must also be provided in the manuscript.

Field-specific reporting

Please select the one below that is the best fit for your research. If you are not sure, read the appropriate sections before making your selection.

☒ Life sciences ☐ Behavioural & social sciences ☐ Ecological, evolutionary & environmental sciences

For a reference copy of the document with all sections, see nature.com/documents/nr-reporting-summary-flat.pdf

Life sciences study design

All studies must disclose on these points even when the disclosure is negative.

Sample size	For electrophysiological and immunohistochemical analyses, sample sizes were determined using effect sizes estimated from pilot cohorts and previous studies. For slice electrophysiological studies, sample size was determined using pilot experiments and prior studies, with n>=11 and N>=3 per group sufficient for a power of >=80%.<br><br>For single-nucleus RNA-sequencing experiments, sample size of 4 mice was determined by a power analysis using effect sizes estimated from our previous studies and a literature search.
Data exclusions	No data were excluded.
Replication	All datasets included 3 or more biological replicates. The snRNA-seq study was done in one cohort of mice.

Randomization	Mice were randomly allocated to groups for all immunohistochemical and electrophysiological studies.
Blinding	Investigators were blinded to all mouse genotype groups during data collection and data analyses for all immunohistochemical and electrophysiological studies.<br><br>Investigators were not blinded during analysis of the single-nucleus RNA-sequencing datasets, as sample metadata (such as mouse genotype groups) was needed to conduct quality control and comparisons.

Reporting for specific materials, systems and methods

We require information from authors about some types of materials, experimental systems and methods used in many studies. Here, indicate whether each material, system or method listed is relevant to your study. If you are not sure if a list item applies to your research, read the appropriate section before selecting a response.

Materials & experimental systems

n/a	Involved in the study	
☐	☒	Antibodies
☒	☐	Eukaryotic cell lines
☒	☐	Palaeontology and archaeology
☐	☒	Animals and other organisms
☒	☐	Clinical data
☒	☐	Dual use research of concern
☒	☐	Plants

Methods

n/a	Involved in the study	
☒	☐	ChIP-seq
☒	☐	Flow cytometry
☒	☐	MRI-based neuroimaging

Antibodies

Antibodies used	Guinea Pig anti-NeuN (1:500), MilliporeSigma, #ABN90<br>DAPI (1:20,000), Thermofisher, #62248<br>Donkey anti-guinea pig 594 (1:1000), Jackson Immuno, #706-585-148<br>Donkey anti-guinea pig 405 (1:250), Jackson Immuno, #706-475-148<br>Donkey anti-rabbit 594 (1:250), Abcam, #ab150076<br>Rabbit anti-mCherry (1:1000), Abcam # ab167453
Validation	Guinea Pig anti-NeuN, validated for IHC in mouse tissue<br>Donkey anti-guinea pig 594, validated for IHC in mouse tissue<br>DAPI, validated for IHC in mouse tissue<br>Donkey anti-guinea pig, validated for IHC in mouse tissue<br>Donkey anti-rabbit, validated for IHC in mouse tissue<br>Rabbit anti-mCherry, validated for IHC in mouse tissue

Animals and other research organisms

Policy information about studies involving animals; ARRIVE guidelines recommended for reporting animal research, and Sex and Gender in Research

Laboratory animals	E3-KI: B6.129P2-Apoetm2(APOE*3)Mae N8, Taconic Biosciences, #1548<br>E4-KI: B6.129P2-Apoetm3(APOE*4)Mae N8, Taconic Biosciences, #1549<br><br>ApoE4-fKI: Apoetm3(APOE_i4)Yhg, available from Yadong Huang (Bien-Ly N, Gillespie AK, Walker D, Yoon SY, Huang Y. Reducing human apolipoprotein E levels attenuates age-dependent Aβ accumulation in mutant human amyloid precursor protein transgenic mice. J. Neurosci. 2012 Apr. 4; 32(14)4802-11).<br><br>Syn1-Cre: B6.Cg-Tg(Syn1-Cre)671Jxm/J, The Jackson Laboratory, #003966<br>GFAP-Cre: B6.Cg-Tg(Gfap-cre)73.12Mvs/J, The Jackson Laboratory, #012886<br><br>ApoE4-fKI mice were previously crossed with Syn1-Cre (#003966) and GFAP-cre (#012886) mice to generate fE4/Syn1-Cre and fE4/GFAP-cre mouse lines (Knoferle J, Yoon SY, Walker D, Leung L, Gillsepie AK, Tong LM, Bien-Ly N, Huang Y. Apolipoprotein E4 produced in GABAergic interneurons causes learning and memory deficits in mice. J. Neurosci. 2014 Oct. 15; 34(42)1469-14078).
Wild animals	No wild animals were used in this study.
Reporting on sex	Female mice were used for this study, as females are more susceptible to developing APOE4-induced AD-like pathophysiology, including clearer neuronal, network, and cognitive phenotypes than males (Leung L, Andrews-Zwilling Y, Yoon SY, Jain S, Ring K, Dai J, Wang MM, Tong L, Walker D, Huang Y. Apolipoprotein E4 causes age- and sex-dependent impairments of hilar GABAergic interneurons and learning and memory deficits in mice. PLoS ONE. 2012;7(12):e53569)

Field-collected samples	No field-collected samples were used in this study.
Ethics oversight	All animal experiments were conducted in accordance with the guidelines and regulation of the National Institutes of Health, the University of California, and the Gladstone Institutes under the protocol AN176773. All protocols and procedures followed the guidelines of the Laboratory Animal Resource Center at the University of California, San Francisco (UCSF) and the ethical approval of the UCSF IACUC.

Note that full information on the approval of the study protocol must also be provided in the manuscript.

Plants

Seed stocks	*Report on the source of all seed stocks or other plant material used. If applicable, state the seed stock centre and catalogue number. If plant specimens were collected from the field, describe the collection location, date and sampling procedures.*
Novel plant genotypes	*Describe the methods by which all novel plant genotypes were produced. This includes those generated by transgenic approaches, gene editing, chemical/radiation-based mutagenesis and hybridization. For transgenic lines, describe the transformation method, the number of independent lines analyzed and the generation upon which experiments were performed. For gene-edited lines, describe the editor used, the endogenous sequence targeted for editing, the targeting guide RNA sequence (if applicable) and how the editor was applied.*
Authentication	*Describe any authentication procedures for each seed stock used or novel genotype generated. Describe any experiments used to assess the effect of a mutation and, where applicable, how potential secondary effects (e.g. second site T-DNA insertions, mosiacism, off-target gene editing) were examined.*
